# Anomaly detection method for business processes based on enhanced complex scenario logs

**DOI:** 10.3389/frai.2026.1819095

**Published:** 2026-08-27

**Authors:** Yang Song, Ruoyuan Zhang

**Affiliations:** 1 Anhui Water Conservancy Technical College, Hefei, China; 2 School of Mathematics and Big Data, Anhui University of Science and Technology, Huainan, China

**Keywords:** anomaly detection, business process mining, cross-modal alignment, discrete data, log enhancement

## Abstract

The quality of logs is crucial for the reliability of business process anomaly detection. Deployed information systems are limited by hardware and cannot generate high-quality event logs containing complex scenario information, thus requiring post-enhancement of log quality. During this process, matching the same entities across different modalities is vital. To maximize the matching degree between complex scenarios and text, we propose a two-stage method for enhancing complex scenario logs. In the first stage, cross-modal entity alignment based on image multi-feature mapping using a Vision-Language Pretraining (VLP) model is employed. Local features are extracted from images, and a gated multi-fusion module is used to dynamically fuse the global features and local multi-features of the image. The Information Noise Contrastive Estimation (InfoNCE) loss function is utilized to train and optimize the model, ensuring tight alignment between images and text. Additionally, a multi-feature enhancer is designed to match the same entities between images and text, integrating image feature information into the original logs. In the second stage, a scenario simulation graph model is constructed to effectively fuse discrete sensor data in complex scenarios with logs, supplementing the discrete data that is often overlooked in the scenarios. Finally, the performance of anomaly detection on enhanced event logs is evaluated using eight anomaly detection models on real scenarios and six commonly used event log datasets. The results show that, compared with other event log datasets, enhanced event logs have higher accuracy in business process anomaly detection. Moreover, ablation experiments are conducted on three different levels of enhanced scenario event logs to verify the effectiveness of the method.

## 1 Introduction

In recent years, anomaly detection has emerged as one of the most dynamic research directions in the field of business process mining (Van Der Aalst et al., 2012). Anomalies such as offsets and absences caused by delayed activity execution and external factors are detected (Van Der Aalst, 2020) from a large number of normal process activities. With its ability to deeply deconstruct and dynamically track business processes (Anonymous, 2019), it provides key technical support for organizations to optimize operational efficiency and achieve data-driven decision support (Balducci and Marinova, 2018). However, practical applications in this field face serious challenges (Martin, 2021). Despite the exponential growth of global data, current anomaly detection technology mainly relies on management system structured event logs (Kerpedzhiev et al., 2021). The event log information covers only 10%−20% of the data in the scenario, while the unstructured scenario information, which accounts for 80%−90% of the total data (e.g., manual operation recordings, environmental data, equipment status, etc.), has not been effectively utilized for a long time (Van Der Aa et al., 2018). This structural data imbalance not only leads to “blind zones” in key aspects of anomaly detection but also limits the development of anomaly detection for complex business processes involving personnel activities and environmental interactions.

The rapid development of anomaly detection techniques has always been closely associated with the evolution of data resources. As a typical data-driven research paradigm, the field is highly dependent on the quality and completeness of structured event logs, a characteristic that is particularly evident in the application of mainstream research platforms. The 12 benchmark datasets released by the Business Process Intelligence Challenge (BPIC) during 2011–2021 provide an important research foundation for core tasks such as process model mining, compliance validation, and predictive monitoring by covering business scenarios in multiple domains, such as finance [loan applications (Van Dongen and Borchert, 2018), insurance claims (Van Dongen, 2011), healthcare (diagnostic processes), and government (building permits (Van Dongen, 2015), event management (Polato, 2017)]. Although these datasets have become standard references in the academic community due to their ability to characterize complex processes across industries, they are still essentially limited to structured operation records generated by management systems, with inherent blind spots in integrating data from unstructured scenarios and tracking activities in physical space. This unidimensionality of data reflects the limitations of the traditional process mining research paradigm and highlights the urgent need to expand multimodal data sources. Although current process mining research can access event logs of typical scenarios such as Helpdesk ticket management (Mannhardt, 2017) and hospital billing systems (Hong Tu and Song, 2016), its application boundary is still constrained by the structural imbalance of data types. Taking the manufacturing industry as an example, although the Production dataset (Knoch et al., 2019), as the only publicly available data source in this domain, attempts to reveal process efficacy through multi-product production logs, its sparse data volume (which contains only a limited number of product categories) and the absence of key parameters (e.g., real-time equipment status, records of manual interventions) severely constrain the depth of analysis. This data dilemma is rooted in two major contradictions: on the one hand, manufacturing companies strictly control core process data due to technical confidentiality requirements, resulting in an inverse relationship between the size of available data and the degree of shop-floor automation; on the other hand, existing research overly relies on the standardized logs generated by management systems (e.g., ERP operation logs), ignoring the information on the synergistic scenarios involving personnel, equipment, and the environment in the physical scene.

In complex scenarios of synergy, cross-modal and cross-resource integration are often involved. VLP models (Radford et al., 2021) can effectively capture cross-modal semantic associations by learning rich cross-modal representations from large-scale unlabeled image–text pair data. The powerful cross-modal understanding ability of VLP models can provide precise semantic associations and fine-grained entity recognition for aligning entities of different modalities in log text enhancement. However, in practical applications, research still faces some challenges and limitations. On one hand, although VLP models have demonstrated excellent zero-shot learning capabilities, the task-agnostic objective adopted in the pre-training stage leads to deficiencies in specific task applications. On the other hand, existing methods mostly rely on global feature similarity between modalities, which represents a coarse-grained cross-modal interaction and may fail to capture more fine-grained feature connections. Regarding these issues, the main contributions of this article are as follows:

Although the present validation is conducted in a pump station motor startup scenario, the proposed framework is not limited to this specific domain. It can be applied to other complex process environments where structured event logs are incomplete but visual scene records and timestamped sensor data are available, such as healthcare workflows, logistics operations, and intelligent manufacturing. In such scenarios, the entity categories can be redefined according to domain-specific objects, operators, locations, and environmental variables, while the two-stage enhancement principle remains applicable.

(1) By learning effective intermediate representations from image features to align with log text features, the representational potential of VLP models can be fully exploited. Based on the predefined task target image features in business process scenarios, a multi-feature enhancer was designed to achieve fine-grained image feature extraction and fusion. This effectively overcomes the difficulty of cross-modal semantic alignment between multimodal data and enhances the log with scene video image data, improving the anomaly detection capability to identify scene anomalies.

(2) A graph model of business processes and discrete data in the scene was constructed to analyze the correlations between business process events and discrete data events in the management system. The graph model was reconstructed to incorporate discrete events into process events, enhancing the correlation between discrete data in the scene and using discrete sensor data to enrich the log, thereby enabling anomaly detection to effectively address the impact of discrete data on business processes in the scene.

(3) Eight popular anomaly detection methods were used to perform trace and attribute types of anomaly detection on the enhanced event logs. Simultaneously, ablation studies were conducted to emphasize the importance of video image data and discrete data for business process anomaly detection. The effectiveness of the proposed method in complex scenarios with limited information capture capabilities of the management system was verified.

In Section 2, the related work in computer vision and scene data for business process mining is reviewed, and the main technical route of the two-stage complex scene log enhancement method is introduced. Sections 3 and 4 describe the cross-modal alignment of image–text data and the multi-feature enhancer, which extracts video image information to enhance event logs. Section 5 explains the method of constructing a graph model to associate discrete data. Section 6 primarily includes anomaly detection experiments and ablation studies, comparing the performance of eight anomaly detection models on event log datasets and enhanced datasets. Finally, the work is summarized in Section 7.

## 2 Related work

In the development of business process mining, Zou et al. (2024) proposed a multimodal activity recognition method that utilizes multiple information modalities (action, sound, environment, and others) captured by IoT sensors in alignment (Rebmann et al., 2019). This leads to more accurate recognition of activities in manual processes and enables the resolution of issues found in these processes. Although it is possible to collect information from the scene, this approach heavily depends on the distribution of hardware devices. Masi et al. proposed a method for using computers to capture business process information from video, addressing the problem of incomplete and inaccurate manual assembly process data (Masi et al., 2018). Meanwhile, Knoch et al. advanced the visual analysis dimension by developing an unsupervised trace modeling framework to extract 3D action topology maps from assembly videos. They achieved compliance comparisons between unstructured operations and standard processes through temporal pattern matching, improving accuracy (Knoch et al., 2020) in identifying process deviations. In the study by Lepsien et al., multiple techniques process video data and propose a method to extract event logs and process models from this data. The results demonstrate that meaningful event logs can be extracted, performing well in terms of stability and significance of the results (Lepsien et al., 2023). Kratsch et al. have made key advances in this area. They presented the first systematic application of computer vision for process mining, successfully capturing details of manual operations that traditional logs often miss through a visual feature extraction module (Kratsch et al., 2022). Their research overcomes the limitation of relying solely on management system data sources. However, by focusing only on specific manual assembly work environments, they overlooked the impact of external assembly scenarios on the operational process. The exclusive reliance on computer vision data fails to establish a connection with external scene data and discrete data from sensors for collaborative analysis. As a result, the information disconnect between the physical space and the management system has not been fully bridged.

**Figure 1:**
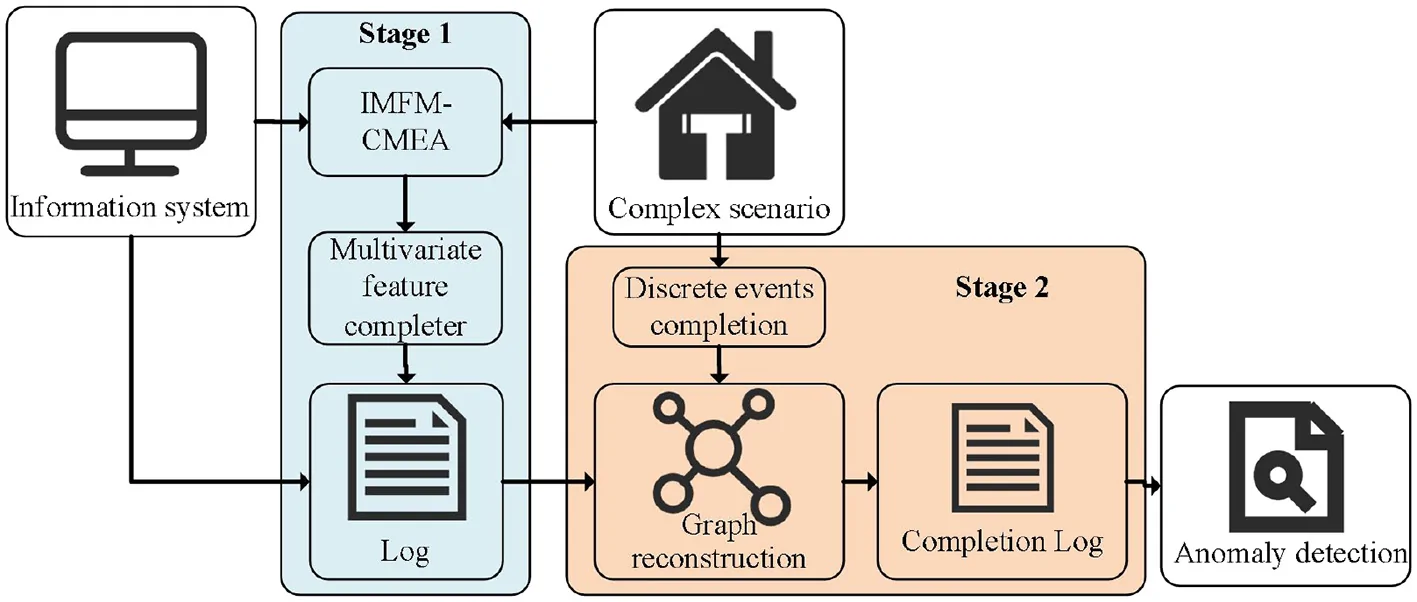
Structure of the scenario log enhancement method.

In actual complex scenarios, critical activities such as operators' on-site decision-making and emergency responses in case of equipment abnormalities are often not recorded by the management system. This leads to systematic deviations between process mining results and actual business execution (Kecht et al., 2021). At the same time, the existing management system, which is designed to form a “data closed loop,” further exacerbates information fragmentation by ignoring the impact of discrete information changes on process development, thus significantly constraining the accuracy and effectiveness of anomaly detection (Raso et al., 2018). This technical bottleneck has seriously hindered the in-depth application of business process anomaly detection in various complex scenarios (Van Eck et al., 2016). Therefore, new methods for scene data perception need to be developed. Although our current research primarily focuses on validating the proposed data perception and enhancement method within a specific industrial pump station scenario, it aims to serve as a preliminary exploration for addressing similar structural data imbalances in broader fields. Existing scene information perception methods often rely on the complementary nature of existing hardware systems and already identified continuous event information. While research results have demonstrated the effectiveness of these methods for business process anomaly detection, they either ignore scene information or discrete events. These methods consider only a single source of information, and the two that are not considered together fail to complement each other, resulting in the loss of potential information. Therefore, this study proposes a business process anomaly detection method based on the enhancement of complex scenario logs. As shown in Figure 1, in the first stage, we propose a method that effectively focuses on both scene information and discrete event information. The Cross-Modal Entity Alignment method based on Image Multi-Feature Mapping (IMFM-CMEA) of the VLP model, by learning effective intermediate representations from image features to align text features, can fully exploit the representation potential of the VLP model and improve its cross-modal recognition accuracy of scene features. The multi-feature enhancer is used to fuse feature information from video images into the original logs, enhancing the logs with scene video images. In the second stage, a log graph model enhancement method is constructed to model the discrete data graph and associate it with the business process model, using discrete data to enhance the logs. Finally, the business process anomaly detection is carried out on the enhanced event logs.

Recently, multimodal data fusion and cross-modal alignment have shown great potential in addressing challenges related to unstructured data in complex scenarios. For instance, Costanzino et al. (2024) explored cross-modal feature mapping for multimodal industrial anomaly detection, demonstrating the efficacy of aligning diverse sensor modalities. Similarly, McKinney et al. (2025) proposed an unsupervised multimodal fusion approach for in-process sensor data to enhance advanced manufacturing monitoring. In the medical domain, Zafar et al. (2026) evaluated visual grounding in multimodal reasoning, highlighting the importance of fine-grained entity understanding. While these state-of-the-art approaches provide valuable insights into cross-modal interactions, they primarily focus on general defect detection or diagnostic reasoning rather than business process step validation. In contrast, our proposed IMFM-CMEA method specifically tailors cross-modal entity alignment (combining Tool, Operator, and Position) to reconstruct and enhance structured business process event logs from unstructured visual scenes.

From the perspective of cross-domain applicability, the key requirement of the proposed method is not the pump station scenario itself but the coexistence of incomplete management-system logs, observable scene entities, and timestamped contextual data. Therefore, similar data enhancement logic can be applied to domains such as medical treatment processes, logistics sorting and dispatching, and manual or semi-automated manufacturing workflows. However, each domain requires redefining the visual entities, event attributes, and sensor variables before empirical deployment.

## 3 Business process scenarios

In complex scenarios, there is often a lack of hardware device support for the management system to effectively detect scene information. The logs generated by some scenarios with management systems only contain the information that sensors can capture, and the data in the unmonitored areas also impact the effectiveness of anomaly detection. In response to this situation, this study takes the pump station startup process as the background. The structured process log information recorded by the original management system in this process only contains simple activity sequences and timestamps, which is not conducive to developing business process anomaly detection. Based on the original management system log data, computer vision technology is used to collect human activity information from the scene video images, enriching the available attribute content of the process logs. An event log dataset that can be relied upon for anomaly detection is constructed to further improve the performance of process anomaly detection efforts. To discover effective activity information from the pump station startup process, the content of the startup process is analyzed and interpreted. According to the management system logs and the “Pump Station Startup and Shutdown Process” manual, the business process activities that need to be executed during the pump station startup process are summarized.

**Figure 2:**
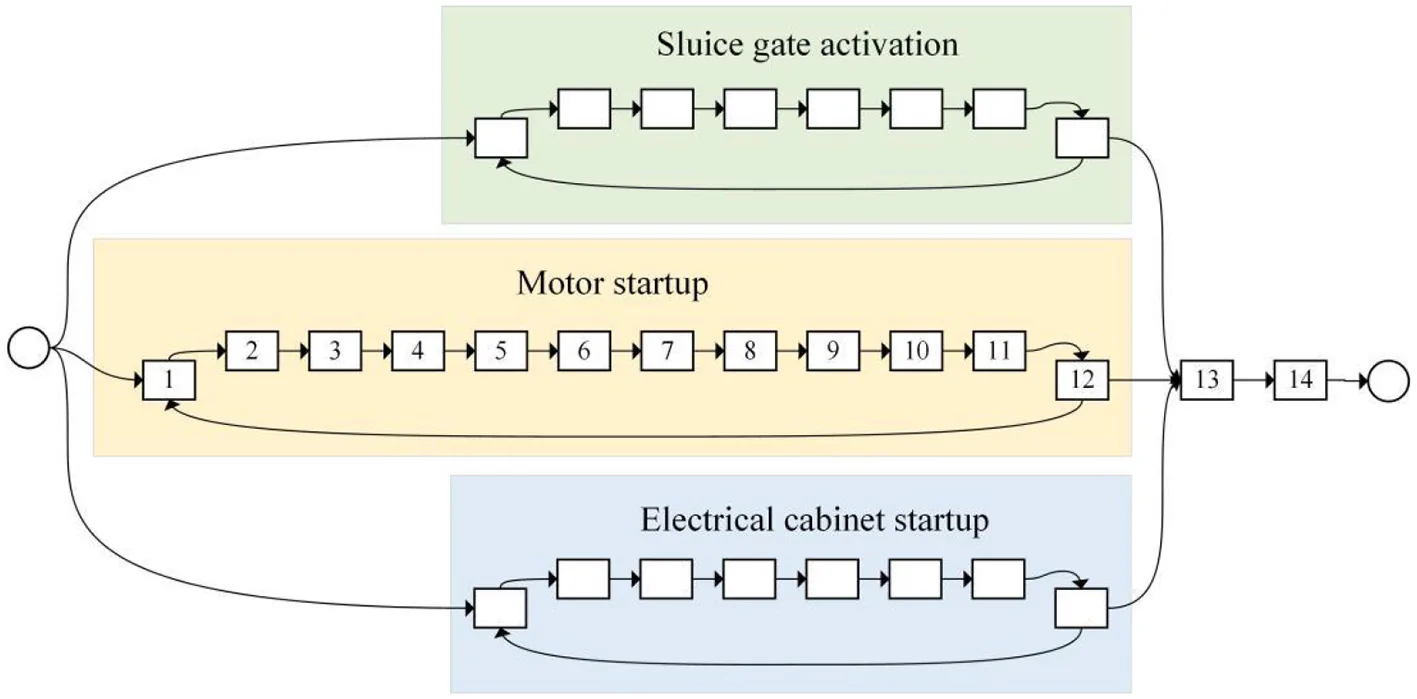
Motor startup scenario business process in the form of Petri net.

The pump station startup process is divided into three scenarios: sluice gate activation, motor startup, and electrical startup. These scenarios are arranged concurrently and executed in sequence. After being enhanced, they are a*ggregate*d to carry out the pump station startup work. Figure 2 below presents the pump station startup process diagram in the form of a Petri net, derived from mining the management system event log using Prom. According to the manual, only one operator is involved in each scenario during the startup process, and the sequence of work activities is fixed. If an abnormality occurs during the task, the pump station startup process is terminated. Therefore, Figure 2 shows only a sequential structure that includes concurrency and loops. Through the Petri net, it can be seen that the motor startup scenario is the most important and contains the most activities in the pump station startup process. Consequently, this study focuses on the motor startup scenario for method research. Table 1 shows the business process of the motor startup scenario in the pump station startup process, with each activity number corresponding to the Petri net activity number.

**Table 1:** Correspondence between activity number and activity content.

Id	Activity
1	Wear protective equipment
2	Check wire grounding
3	Remove coupling guard
4	Check coupling
5	Check drive shaft
6	Install coupling guard
7	Check gauges
8	Turn on the power
9	Adjust flow rate
10	Checking the operating status
11	Check motor temperature
12	Organize the site
13	System-wide confirmation
14	Perform startup procedure

### 3.1 Selection of target features

Videos are formed by continuously stacking image frames and splicing them in chronological order. Frames are the smallest units of a video and the primary objects for content recognition. The main task of the data preprocessing module is to extract key content information, or features, from the complete video stream, which typically reflects the main content of an activity. In a task, the frame with the highest content similarity is selected as the key frame, and the number of key frames depends on the complexity of the activity. As a medium for information transmission, videos are composed of continuous or discontinuous image sequences. With the advancement of technology, parameters such as resolution, bit rate, and frame rate have been continuously improved to enhance visual quality, which has also led to a large amount of redundant information in videos. Based on the 14 activities in the motor startup scenario, the video content was divided, and videos containing only the 14 activities were selected to filter out unnecessary content that could affect the quality of log construction.

**Figure 3:**
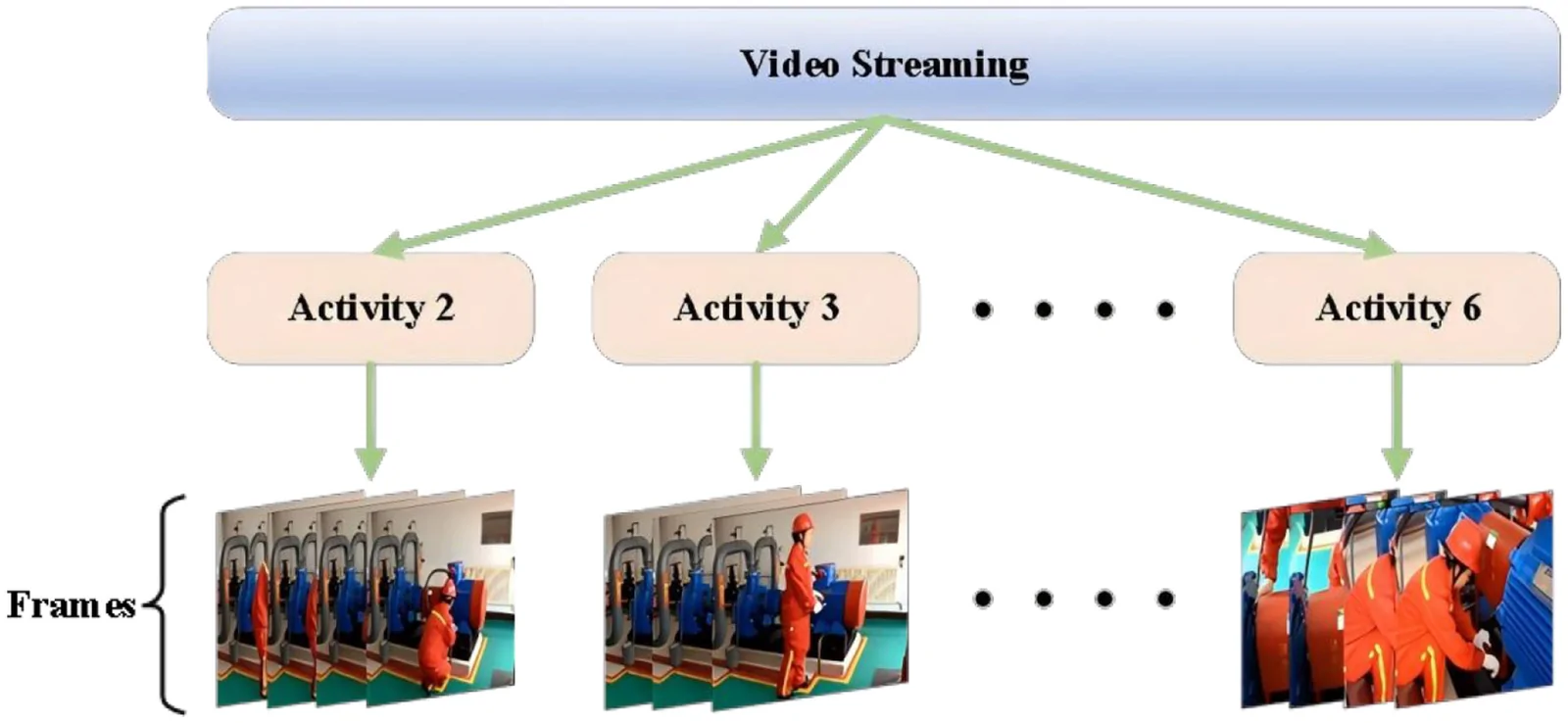
Composition of the video.

Figure 3 shows the video composition of the “Motor startup” scene in the pump station startup process. During this process, the operator must perform multiple activities in sequence to ensure the normal startup and operation of the motor. These tasks are executed in accordance with the Petri net process diagram in Figure 1. There is a temporal relationship between the preceding and subsequent tasks, and the video stream is segmented based on the content of the activities. The start and end times of an activity are defined as the beginning and end of the task, respectively, and the video stream is segmented into individual parts that are independent but have a sequential relationship with one another. For example, activity 2 (Wear protective equipment) occurs before activity 3 (Remove coupling guard), and the transitional video stream between activities 2 and 3 includes complex information such as the operator's movement and tool switching. The loss of this information would seriously affect the quality of the event log; therefore, we focus on the video information related to the execution of activities. Key frames of the video are sampled in chronological order. A high sampling frequency requires more advanced hardware for the equipment, while a low sampling frequency may lead to the loss of video content, thereby affecting the quality of subsequent logs. Considering the execution cost, a suitable frequency must be selected. According to the framework proposed in the literature (Kratsch et al., 2022), the sampling rate of the video stream is set to 1 frame per second, and the video stream of the current activity is sampled once per second.

Typically, video images contain a wealth of target information. To extract the effective information related to activities from them, it is necessary to first label the features of the targets and then identify each target in the video images. According to the regulations in the “Pump Station Startup and Shutdown Process” manual, the items needed for the “Motor Startup” scene activities are labeled with features. The targets in the pump station startup process are categorized into three types: Tool, Operator, and Position. Tool records the tools used in the activity, Operator records the individual performing the activity, and Position records the location of the operator during the activity. These categories also include specific targets as follows:

**Tools**: Helmet, gloves, adjustable wrench, steel rule, electroprobe, insulated gloves, flat screwdriver, and thermometer;

**Operator**: The operator information;

**Position**: The location of the operator performing each activity.

The three types of targets in the video image are labeled as the required features, which are key factors affecting the normal execution of the motor startup scenario. There are quality requirements for the three types of target features: tool selection, operation execution position, and operator identity during the activity. Abnormal information in the activity can cause safety hazards, so these factors are regarded as the essential information that needs to be obtained from the scene. Why are these features selected? The following three examples explain these features.

Example 1: During the activity, the operator is required to use tools in accordance with safety requirements; incorrect usage could lead to a safety incident. However, in Activity 5 (Check Drive Shaft), gloves should not be used because it is necessary to use the hands to turn the drive shaft, and wearing gloves can lead to injuries from hand dragging. Thus, the correct use of tools as required in the corresponding activity is essential for ensuring the safe operation of the process.

Example 2: During the startup of a pumping station, the operator must be certified as a licensed operator, as unauthorized and unqualified personnel performing the activity may lead to equipment failures. Additionally, during Activity 8 (Turn on the power), the operator should be the only person on site, as the presence of other personnel may result in electrocution.

Example 3: Each activity has a designated location for performing the operation, and as the activities are performed in sequence, the locations change in order. Incorrectly positioned operations may pose a risk of potential process errors.

**Figure 4:**
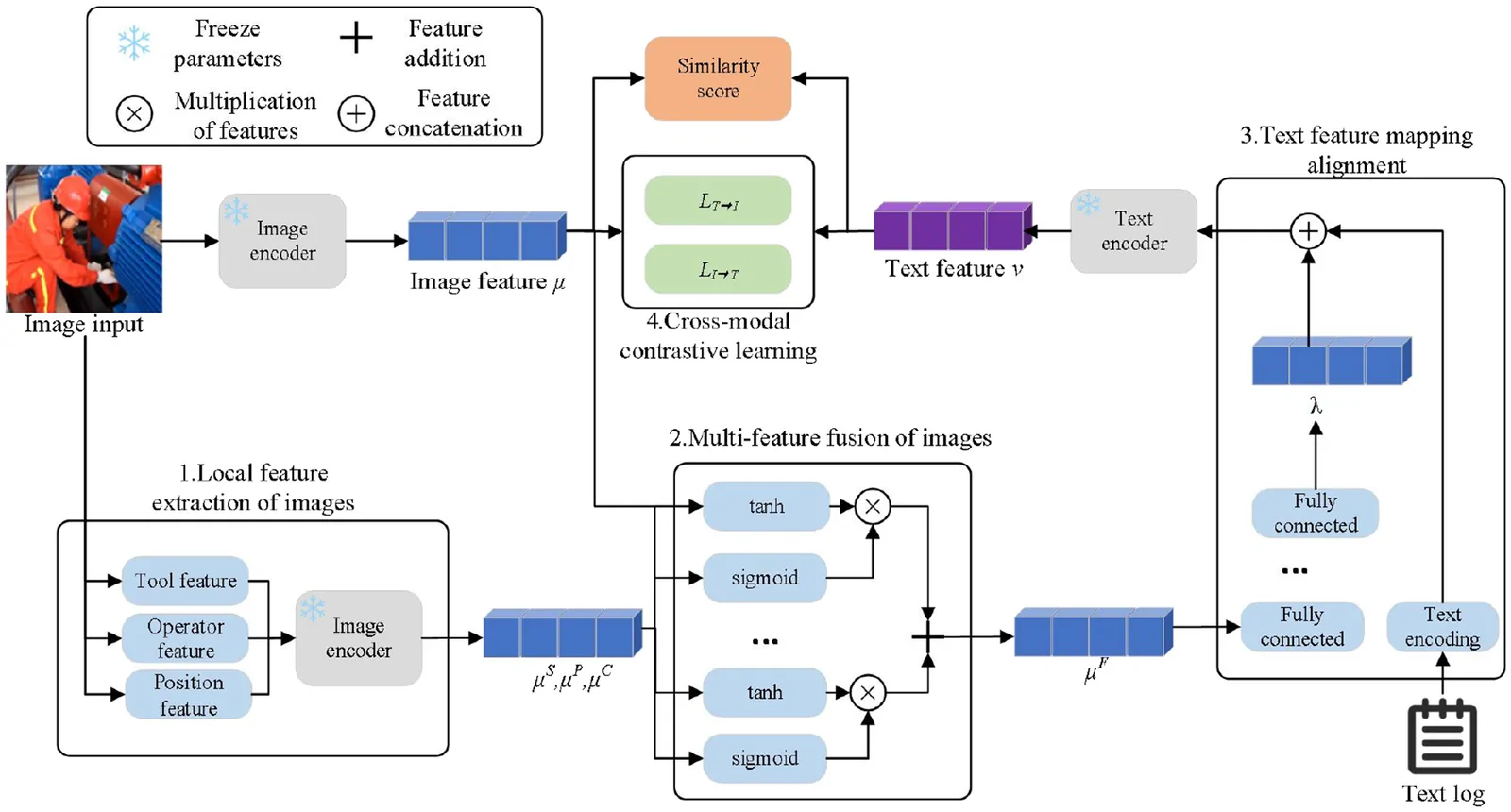
The structural diagram of the IMFM-CMEA.

Based on the content of the scenario, the entire motor startup scenario is presented as shown in Figure 4. In this scenario, the operator executes Activity 1 to start, and each activity requires a different tool and occurs at a different location. Based on this scenario activity, the attributes of the events corresponding to each activity are captured. The operator enters the scenario, starts from position A, and uses the tool to execute the activity.

### 3.2 IMFM-CMEA

Aligning the features of image and text logs in a unified feature space is the core of cross-modal entity alignment tasks. This study proposes a method based on VLP models, namely the Image Multi-Feature Mapping Cross-Modal Entity Alignment method (IMFM-CMEA). The overall structure of IMFM-CMEA is shown in Figure 4. The image and text encoders are initialized with publicly available VLP models, and the parameters of the encoders remain unchanged.

The IMFM-CMEA mainly consists of four modules: (1) Image Local Multi-Feature Extraction: Based on the visual characteristics of celadon images, local features in terms of contour, texture, and color are extracted from the image. The image encoder then generates the corresponding local features μ^S^, μ^P^, and μ^C^; (2) Image Multi-feature Fusion: The gated multi-fusion unit adaptively allocates weights to the input image feature μ and the local multi-features μ^S^, μ^P^, and μ^C^ to generate a reliable fused feature; (3) Text Feature Mapping Reconstruction: First, the input fused feature μ^F^ is transformed into a mapped feature λ through a multi-layer fully connected mapper. The encoded text is then fused λ and input into the text encoder to align the text feature *v*more closely with the matching image feature μ; (4) Cross-modal Contrastive Learning: The Information Noise Contrastive Estimation (InfoNCE) loss is constructed for the training of IMFM-CMEA and is divided into two parts: *L*_*T* → *I*_ and *L*_*I* → *T*_. Through similarity calculation, the similarity score between the image and the text can be obtained.

#### 3.2.1 Local multi-feature extraction of images

In IMFM-CMEA, the extraction of multiple image features plays a crucial role. By capturing the image features of Tool, Operator, and Position in the Motor startup scenario, it provides key visual information for the subsequent reconstruction of log text features. This local multi-feature selection strategy can significantly enhance the effective association and alignment of entities across different modalities.

Specifically, for a given image *I*, this study extracts its local features in terms of contour, texture, and color based on digital image processing technology. Algorithm 1 presents the pseudo-code of the extraction process.

Algorithm 1 **Image characteristics extraction.**

**Input**: image ***I***
**Output**: Local multi-feature extraction results
1. *I* ←U2-Net(*I*)
2. *I* ←Processing(*I*)
3. dim *S*,*P*,*C*
4. *S* ←Canny(*I*)
5. *P* ←LBP(*I*)
6. *I* ←HSI(*I*)
7. *C* ←GMMs(*I*)
8. *C* ←RGB(*C*)
9. *ţ*^*S*^, *ţ*^*P*^, *ţ*^*C*^ ← *Image Encoder*(*S*,*P*,*C*)
10. **return** *ţ*^*S*^, *ţ*^*P*^, *ţ*^*C*^

To highlight the most prominent parts of the image, U2-Net is used to segment the image (Qin et al., 2020), delineating the main contours of the target in the scene and initially removing invalid pixels. This constitutes the image preprocessing to enhance image quality. First, Gaussian filtering is applied to smooth the image, reducing random noise and minor fluctuations; then, the contrast of the image is enhanced to make the differences between various parts of the image more obvious and to make the texture and structure more prominent. The Canny operator is then adopted to identify the key structural contours of the main body in the image. The Canny operator is an edge detection algorithm with high accuracy and strong noise resistance. Meanwhile, the local binary pattern, which has strong gray-scale and rotation invariance, is utilized to capture the texture information of the main body. Next, the Gaussian Mixture Model (GMM) is employed to extract color features (Li et al., 2018). This model is composed of multiple Gaussian distributions and is trained through the expectation–maximization algorithm, which can adapt to complex data distribution patterns. Before extracting the target features, the image is first converted to the hue–saturation–intensity (HSI) color space, as this space is closer to human perception of color and provides a more intuitive color representation than red–green–blue (RGB).

**Figure 5:**
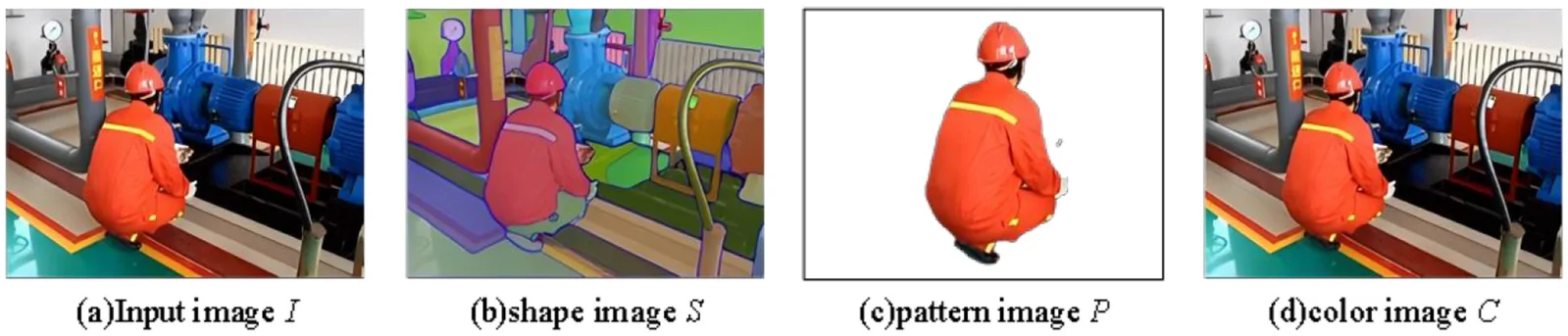
Visualization results of local multi-feature extraction. **(a)** Input image *I*, **(b)** shape image *S*, **(c)** pattern image *P*, and **(d)** color image *C*.

Figure 5 shows the visualization results of the local features extracted from the input image in Figure 5a, specifically the target feature shape in Figure 5b, the pattern in Figure 5c, and the color in Figure 5d. The final image encoder converts all images into the corresponding features, μ^P^, and μ^C^.

#### 3.2.2 Multi-feature fusion of images

The multi-feature fusion strategy of the image is inspired by literature (Arevalo et al., 2020), adopting a feature fusion strategy based on gating. During the training process, this strategy automatically assigns weights to each feature without manual adjustments. Specifically, the gated multi-fusioner first learns the appropriate fusion weights for each feature to integrate multiple features μ^S^, μ^P^, and μ^C^ of the given image. The Equations 1, 2 are calculated as follows:


$$\begin{array}{l} \mu^{\prime }\text{=}LN_{0}\left(\mu \right)⊕LN_{1}\left(\mu^{S}\right)⊕LN_{2}\left(\mu^{P}\right)⊕LN_{3}\left(\mu^{C}\right) \end{array}$$ (1)

$$\begin{array}{l} z_{i}=\sigma \left(LN_{i}\left(\mu^{\prime }\right)\right) \end{array}$$ (2)

In the formula, μ′ represents the initial integration result of multiple features of the image; *LN* denotes the linear layer; ⊕ indicates the concatenation operation; *z*_*i*_ represents the fusion weight corresponding to each image feature, where *i* ∈ [0, 3] and *z*_*i*_ ∈ [0, 1]; and σ represents the sigmoid activation function.

Finally, when performing feature fusion, the proportion of each image feature is determined by the fusion weight *z*_*i*_, and the Equation 3 are calculated as follows:


$$\begin{array}{l} \mu^{F}=\overset{M}{\underset{i=0}{\sum }}\theta_{i}\left(LN_{i}\left(\mu_{i}\right)\right)\times z_{i} \end{array}$$ (3)

In the formula, μ^F^ represents the fusion result of multiple features of the image; *M* indicates the number of features to be fused, with *M* ∈ [0, 3]; θ represents the tanh activation function; μ_*i*_ represents the features to be fused, namely μ, μ^S^, μ^P^, and μ^C^.

#### 3.2.3 Log text feature mapping alignment

By using image features to guide the alignment of text features ν, it can be aligned with image features μ. First, a multi-layer fully connected mapper is designed to transform the fused feature μ^F^ into a mapped feature λ, and the transformation process Equation 4 are calculated as follows:


$$\begin{array}{l} \lambda =\varphi \left(\eta \left(LN\left(l,m,o,\mu^{\text{F}}\right)\right)\right) \end{array}$$ (4)

In the formula, λ represents the mapping feature; φ represents the rectified linear unit (ReLU) activation function, with no ReLU in the output layer; η represents the dropout regularization technique, with no dropout in the output layer; *l* indicates the number of fully connected propagation layers; *m* represents the number of hidden neurons in the intermediate layer; and *o* indicates the output dimension of the mapping feature.

Next, the mapping feature λ is fused with the encoded text *T*, and then it is input into the text encoder. The Equation 5 are calculated as follows:


$$\begin{array}{l} v=f\left(\psi \left(T\right)⊕\left(s,\lambda \right)\right) \end{array}$$ (5)

In the formula, *v* represents the aligned text features; *f*() represents the text encoder; ψ represents the Tokenizer text encoding; *T* represents the text; and *s* represents the scaling range of the mapped feature λ.

#### 3.2.4 Cross-modal contrastive learning

The InfoNCE loss function is used to optimize the learning results of the model for cross-modal contrastive learning. During the training process, the connection between the image feature μ and the text feature is established. The similarity between them is measured by calculating the cosine similarity between cross-modal features. First, the contrastive loss *L*_*T* → *I*_ from text to image is calculated, and the Equation 6 are calculated as follows:


$$\begin{array}{l} L_{T\to I}=-\frac{1}{N}\overset{N}{\underset{i=1}{\sum }}\log\frac{\exp\left(v_{i}^{T}\mu_{i}\xi \right)}{\sum_{j=1}^{N}\exp\left(v_{i}^{T}\mu_{j}\xi \right)} \end{array}$$ (6)

In the formula, *N* represents the number of image–text pairs; *v*_*i*_ and *v*_*i*_ denote positive sample pairs; *v*_*i*_ and μ_*j*_ denote negative sample pairs; and ξ ∈ *R* represents a learnable temperature coefficient.

Next, calculate the image–text contrastive loss, *L*_*I* → *T*_ and the Equation 7 are calculated as follows:


$$\begin{array}{l} L_{I\to T}=-\frac{1}{N}\overset{N}{\underset{i=1}{\sum }}\log\frac{\exp\left(v_{i}^{T}\mu_{i}\tau \right)}{\overset{N}{\underset{j=1}{\sum }}\exp\left(v_{i}^{T}\mu_{j}\tau \right)} \end{array}$$ (7)

Finally, by combining Equations 6, 7, the final contrastive loss *L*_*I*↔*T*_ of the model is obtained, with the Equation 8 are calculated as follows:


$$\begin{array}{l} L_{I↔T}=\frac{1}{2}\left(L_{T\to I}+L_{I\to T}\right) \end{array}$$ (8)

### 3.3 Multivariate feature completer

Align the video image features and text log features in the scenarios mentioned above. After alignment, the recognized image features need to be saved and integrated into the original log text. Design a multi-feature enhancer to perceive, extract, and fuse the video image features. Describe the information in the current video image in the form of text data, and store all the extracted data using a storage method similar to an array.

In the scene process section, the original logs of the management system are used for Petri net modeling. To better supplement the scene information of the logs, it is necessary to standardize the definition of the logs. Referring to the temporal relationship of the activities in the Petri net formal representation of the pump station startup process diagram mentioned above, the sequence of activities is divided. Each activity is an event, and the information array corresponding to the activity in the video image is the attribute of the event. Based on this, the logs are defined.

**Definition 1 (process)** A process is a series of activities that have a back-and-forth relationship and interact to achieve a specific goal.

**Definition 2 (event)** An event records information about an activity and the attributes contained within the activity during the startup execution of a pumping station. For example, an event that checks the “motor wiring and grounding” operation has five arrays of attributes, including *Tool, Operator, Position, Timestamp*, and *Num*. Each act is an activity in the operation, *Start_time* and *End_time* are the start and end times of the activity, and *Consuming_time* is the duration of the activity. *Tool* is the tool needed for the activity, *Operator* is the identity of the operator, and *Position* is the location of the operator during the activity.

**Definition 3 (case)** A case is a sequential name for a set of events with the same ***case***, which can be regarded as the process of a complete sample. It is denoted as δ = {*e*_1_, *e*_2_, ⋯ , *e*_*n*_}(*n* ≥ 1), where is the *i*(1 < *i* ≤ *n*)−thevent, and*n* is the total number of events in the case.

**Definition 4 (trace)** A trace represents *j*the ordered events in a case and can be expressed as δ_*j*_ = {*s*_1_, *s*_2_, ⋯ , *s*_*j*_}.

**Definition 5 (event log)** An event log is a set of cases, *L* = {δ|δ ∈ *U*, 1 ≤ *i* ≤ *N*}, where *U* denotes the set of all cases, and *N* is the number of cases in the event log *L*.

**Figure 6:**
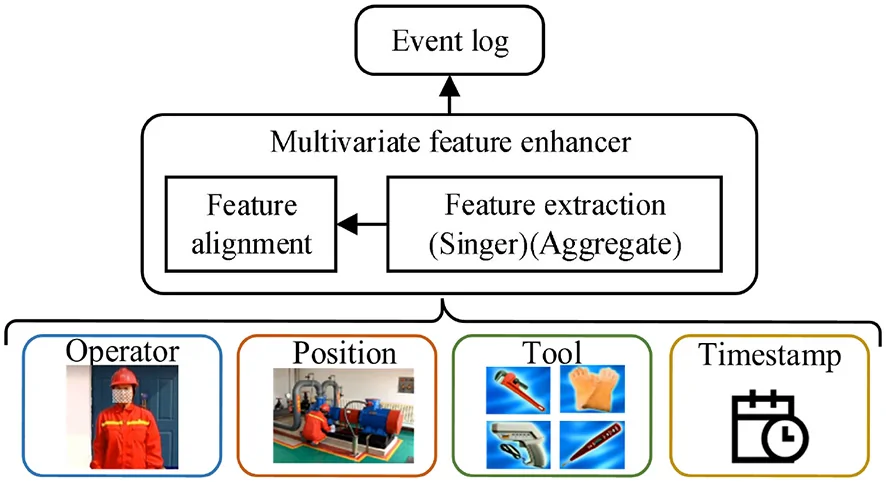
Structure of the Multivariate Feature Enhancer.

In this study, the scene video data is parsed and labeled, and the *Tool, Operator, Position, Num*, and *Timestamp* arrays are defined to store the corresponding data. Based on this premise, a feature extraction method is designed to extract Tool, Operator, Position, Num, and Timestamp information from the video images. According to the three types of targets in the video image content, the arrays *Tool, Operator*, and *Position* are used to store the extracted scene features, with each meta-feature of the Multivariate Feature Enhancer stored in its corresponding array. A *Timestamp* array and a *Num* array are also required; the *Timestamp* records the start and end times of the video images detected by the target's location within the entire video stream, while the *Num* records information about the number of Tools and Operators in each activity. Figure 6 shows the structure of the Multivariate Feature Enhancer.

To define and enhance five image feature data arrays, we designed two different types of feature extractors: the *Singer* extractor and the *Aggregate* extractor, which are used to extract different types of video image data.

Extractor *Singer*: extracts *Tool, Operato*r, *Positio*n, and *Timestamp* information from the video images.

**Figure 7:**
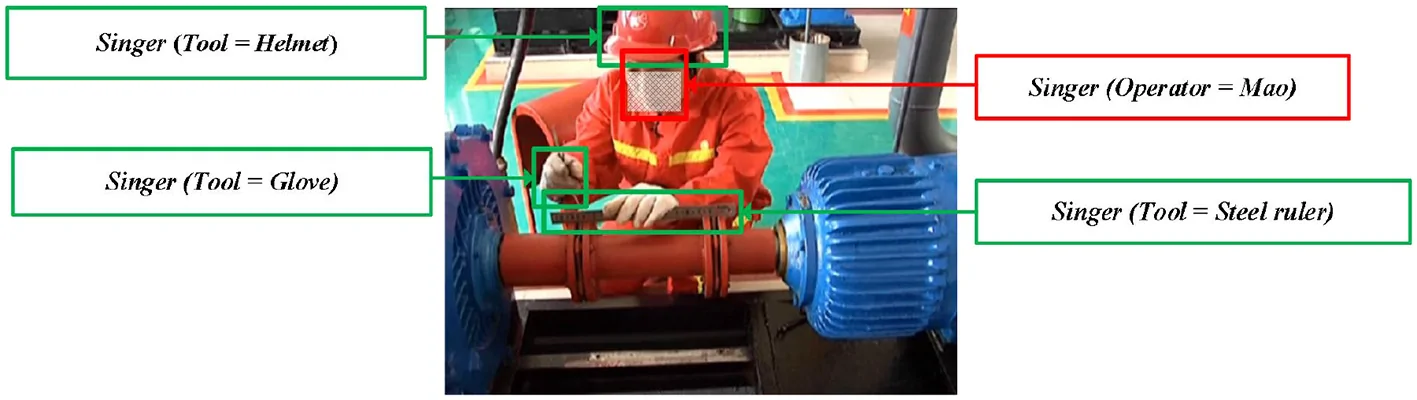
*Singer* feature extractor. Extractor *Aggregate*: extracts *Num* and *Timestamp* information from the video images.

Algorithm 2 describes the process of extracting image features using the *Singer* feature extractor. This method extracts the feature target information from a video image at a time, including the three types and eight operational tool targets specified earlier, as well as the operator and position features, storing the data in four arrays (*Tool, Operator, Position, Timestamp*), respectively. Algorithm 2 outlines the specific execution steps of the method. The input to the algorithm is a video stream, and the output is an array. The algorithm can detect video images and determine whether the feature targets exist in the current video image. If the target does not exist or is not detected, it will return a null value. If the target exists, the result can be returned and stored in a tuple. In Figure 7, it can be observed that the detection boxes of different colors in the video image correspond to different tuples. Green represents the detection box of the *Tool* array, and red represents the detection box of the *Operator* array. In the coupling activity, there are four detection targets: *Helmets, Gloves, Steel rulers*, and *Operators*. Each labeled target has a corresponding detection box. After detecting the *Helmet*, it is identified that the *Helmet* belongs to the *Tool*, and the information about the *Helmet* is stored in the *Tool* array.

Algorithm 2 ***Singer* feature extractor example.**

**Input**: *Video* image ***I***
**Output**: *Tool, Operator, Position, Timestamp*
1. **for** *Video* image ***I***
2. **if** *Tool* **or** *Operator == null* **then**
3. *Tool* ← ‘ ’
4. *Operator* ← ‘ ’
5. *Position* ← ‘ ’
6. *Timestamp* ← ‘ ’
7. **else**
8. *Tool* !*== null* **or** *Operator*!*== null*
9. *Tool* ← ‘*Helmet*’
10. *Operator* ← ‘*Mao*’
11. *Position* ← ‘*B*’
12. *Timestamp* ← *date*()
13. **end**
14. **end**
15. **return** *Tool, Operator, Position, Timestamp*

Algorithm 3 describes the process of an *Aggregate* feature extractor for extracting image features. In this algorithm, there are two arrays, *Num* and *Timestamp*, which record the number of *Singer* feature extractors and the timestamps in video images. The input of the algorithm is the video stream and the four arrays output by the *Singer* extractor mentioned above. For the *Singer* extractors corresponding to the same target, they are matched through timestamps. After matching, *Num* records the number of *Singer* feature extractors present at the same time for the same target, while *Timestamp* records the timestamps of the matched video images. The number of identical *Singer* feature extractors is accumulated, and the counts of *Singer* extractors with different labels are accumulated separately. As shown in Figure 7, there are three *Singer* feature extractors, corresponding to one *Helmet* and two *Gloves*. The *Aggregate* feature extractor counts the *Singer* extractors of *Helmet* and *Glove*, respectively, where *Num* of *Helmet* = 1 and *Glove* = 2. At the same time, the timestamps of these *Singer* feature extractors' video images are recorded.

Algorithm 3 ***Aggregate* feature extractor example.**

**Input**: *Video* image ***I***, *Singer*(*Tool, Operator, Position, Timestamp*)
**Output**: *Num, Timestamp*
1. **for** *Singer*() **do**
2. **if** *Singer.Tool != null* **then**
3. *Num* ← *Singer.Tool.Helmet.count*()
4. *Timestamp* ← *Singer.Tool.Helmet.date*()
5. **else**
6. *Singer.Tool == null;*
7. *Num* ←' ';
8. *Timestamp* ←' ';
9. **end**
10. **end**
11. **return** (*Num,Timestamp*)

The definitions of process, event, case, trace, and event log are explained. Once the log definitions are completed, the start and end times of each event need to be specified, using Algorithm 4 to complete the timestamp process.

Algorithm 4 **Time generation.**

**Input**: *Singer*(), *Timestamp*()
**Output**: *Start time, End time, Consuming time*
1. **if** *Timestamp != null and Singer*() *!= null*
2. *Start time* ← *Timestamp.st*
3. *End time* ← *Timestamp.et*
4. *Consuming time* ← *et-st*
5. **end**
6. **return** *Start time, End time, Consuming time*

Algorithm 4 is a process of time extraction and alignment, with the input being activities and timestamps, and the output being the start time, end time, and duration of the activities. If the activity exists in the video stream and the timestamps of the activity video images are correctly recorded, three time points will be assigned (see line 1 of the algorithm). The time of the video image when the activity is detected to start is the start time of the activity, and similarly, the time of the video image when the activity ends is the end time of the activity, while the time period between the start and end of the activity is the duration of the activity (see lines 2 to 4 of the algorithm).

**Figure 8:**
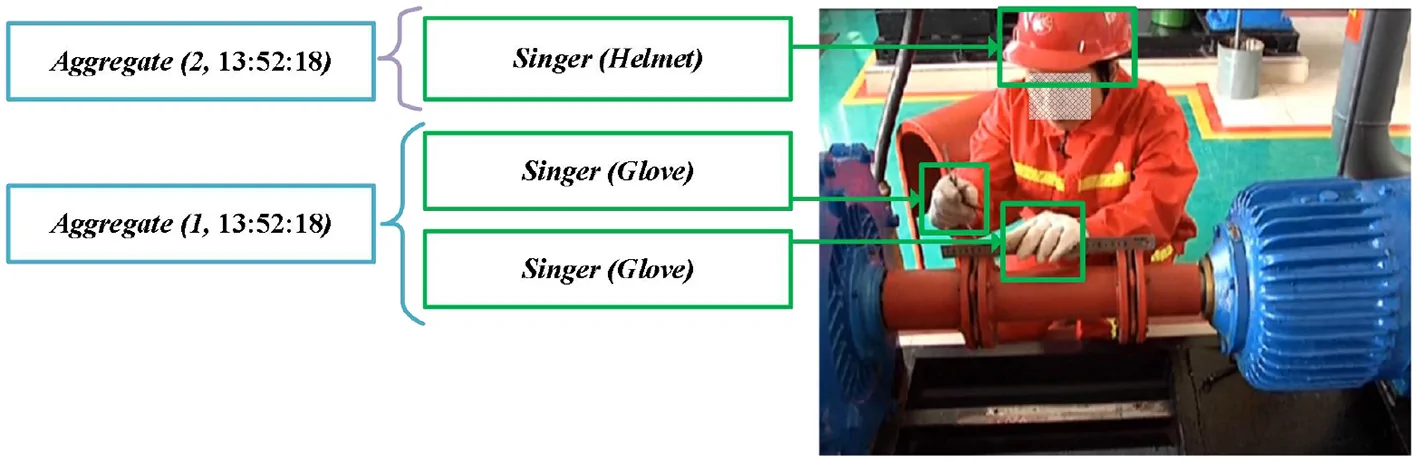
*Aggregate* feature extractor.

Algorithm 5 is the attribute assignment and log enhancement method. In the log enhancement process, each upcoming operation is treated as an event, and various attribute values corresponding to each event are assigned. The inputs to the algorithm are Video Stream, *Singer*, and *Aggregate*, while the output is the event log *L*. The first step is to ensure that there is an input of video streaming data and that the video images are sampled at a frequency of one frame per second. Since some activities may last too long and result in redundant video images, identical activities and content are represented by the same video image, with the length of this type of video image corresponding to the length of the log *N* (see algorithm lines 1 to 2). *Singer* and *Aggregate* work separately; therefore, to match them, both feature extractors have common parameter arrays. If the array parameters are the same, then these two feature extractors are considered matched. For example, in the video images of Figures 7, 8, for extracting protective equipment features, *Singer* identifies two feature targets in the *Tool* array: *Helmet* and *Glove*. *Aggregate* completes the matching of the feature extractors when it finds the same feature target content, additionally recording the number of *Helmet* and *Glove* feature targets (see line 4 of the algorithm). The target features of the video images are then assigned to the defined log attributes, where *case* represents the execution number, *Act* is the name of the activity, and each of the seven attributes corresponds to five target feature values (refer to algorithm lines 7 to 10). The start time, end time, and duration of the activity in the log are assigned values through Algorithm 3 (refer to algorithm line 11). Table 2 presents a complete trace of the enhanced log in the first stage, which records multiple attribute characteristics of each activity during the execution process of the pump station motor startup scenario.

Algorithm 5 **Log completion.**

**Input**: *image I, Singer*(), *Aggregate*()
**Output**: Log*:L*
1. *L*←Ø
2. *N = Length*(*I*)
3. **for** *i* in range(1,*N*) **do**
4. **if** *Singer.Tool* == *Aggregate.Tool*
5. *case* ← *i*
6. *Act* ← *Activity*(*i*)
7. *Tool* ← *Singer.Tool*
8. *Num* ← *Aggregate.Num*
9. *Position* ← *Singer.Position*
10. *Operator* ← *Singer.Position*
11. *Start time, End time, Consuming time* ← *Time generation*()
12. **end**
13. **return** *L*

**Table 2:** Enhanced scene logs in the first stage.

Case	Act	Tool	Num	Position	Operator	S_t	E_t	C_t
e1	Wear protective equipment	Helmet, gloves	2		Mao	13:40:57	13:46:34	0:05:37
e2	Check wire grounding	Helmet, gloves, and adjustable wrench	3	A, E	Mao	13:47:02	13:50:11	0:03:09
e3	Remove coupling guard	Helmet, gloves, and adjustable wrench	3	C, G	Mao	13:50:23	13:51:04	0:00:41
e4	Check coupling	Helmet, gloves, and steel rule	3	C, G	Mao	13:51:17	13:52:18	0:01:01
e5	Check Drive Shaft	Helmet	1	B, F, D	Mao	13:54:23	13:57:11	0:02:48
e6	Install coupling guard	Helmet, gloves, and adjustable wrench	3	C, G	Mao	13:57:43	13:58:56	0:01:13
e7	Check gauges	Helmet, gloves, and electroprobe	3	E	Mao	14:01:07	14:03:46	0:02:39
e8	Turn on the power	Helmet, insulated gloves	2	H	Mao	14:04:27	14:05:13	0:00:46
e9	Adjust flow rate	Helmet, gloves	2		Mao	14:06:02	14:09:32	0:03:30
e10	Checking the operating status	Helmet, gloves, and screwdriver	3		Mao	14:10:02	14:12:43	0:02:41
e11	Check motor temperature	Helmet, gloves, and thermometer	3	A, E	Mao	14:12:56	14:14:12	0:01:16
e12	Organize the site	Helmet, gloves	2		Mao	14:18:03	14:26:53	0:08:50
e13	System-wide confirmation	Helmet, gloves	2		Mao	14:28:34	14:46:05	0:17:31
e14	Perform startup procedure	Helmet, gloves	2		Mao	14:48:13	14:49:23	0:01:10

## 4 Discrete data association

The above work has enhanced the extraction of information from video image data of the scene to enrich the attributes of the event log. However, during the actual pump station startup process, in addition to video data, there is other external scene information that can affect the startup process. For example, additional temperature and vibration sensors installed outside the management system collect information on temperature and vibration in the scene. This information is not directly related to the attributes of event activities, making it difficult to match through simple temporal dependency relationships.

During the pump station startup process, temperature and vibration data can be used as external inputs to assist in identifying process anomalies. However, traditional temperature and vibration anomaly detection primarily involves threshold detection of the data. For instance, the maximum allowable temperature for the pump station motor is 180 °C, and the performance reference temperature is 145 °C. The temperature sensor monitors the motor's temperature in real time. If the temperature falls below 145 °C, a prompt is issued; if it exceeds 180 °C, an alarm is triggered. However, the temperature data during the pump station startup process is only available after activity 8 (start power supply). If temperature data appears before activity 8 and the motor is started without following the proper process steps, it may lead to safety hazards. Traditional temperature monitoring methods cannot detect “temperature errors” in the process, meaning they fail to identify when temperature data appears in activities where it should not. Similarly, traditional vibration anomaly detection focuses solely on waveform data and overlooks potential process errors, leading to abnormal equipment operation.

### 4.1 Graph model construction

**Figure 9:**
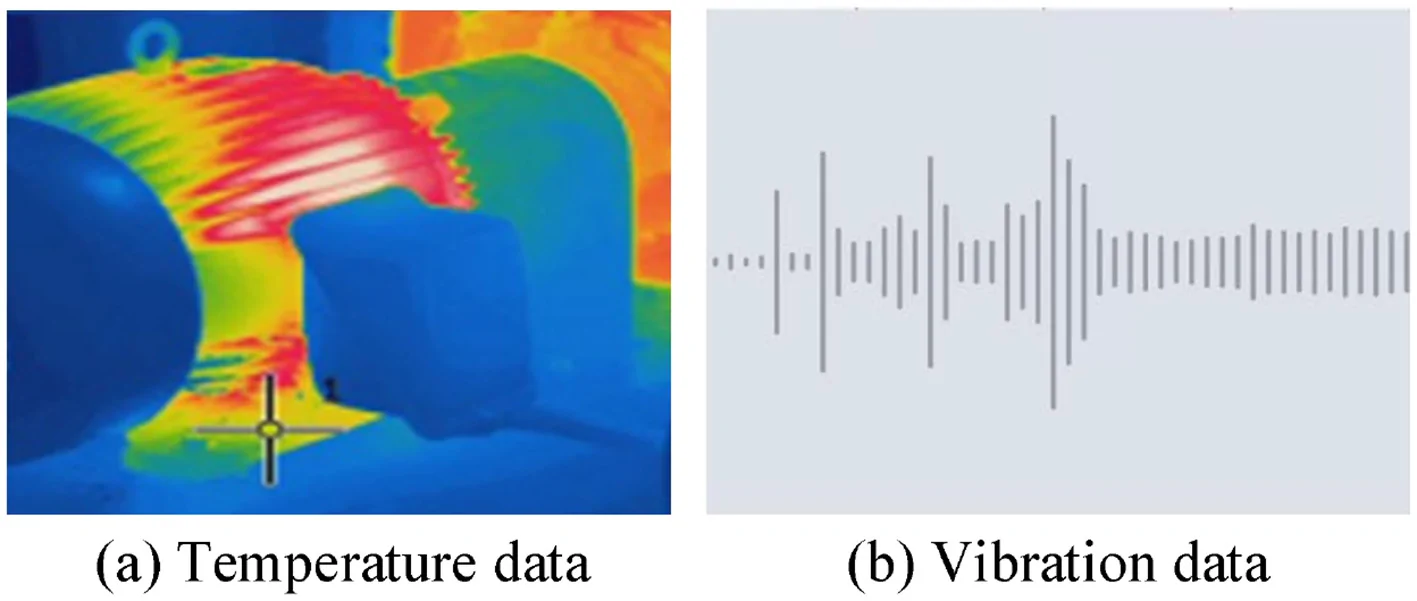
**(a)** Temperature data and **(b)** vibration data in the motor startup scenario.

In the motor startup scenario, temperature and vibration information are enhanced for detection through additional sensors. The management system overlooks these pieces of information, and they cannot be captured from the video either. Therefore, after enhancing the video information, these discrete pieces of data need to be modeled into a graph to enrich the event log's perception of the scene by incorporating temperature and vibration information. The temperature data refers to the temperature of the motor during the pump station startup process. Figure 9a is a schematic diagram of real-time detection of motor temperature using a visual thermometer. The motor temperature can be categorized into three states: the static state, the changing state, and the stable state. Among these three states, the static state corresponds to the temperature of the motor before startup, which is at room temperature. The changing state corresponds to the rapid temperature rise of the motor shortly after startup. When the temperature stabilizes and no longer fluctuates, it enters the stable state. Since the changing state is brief and the temperature threshold exists between the static and stable states, the changing state is disregarded. The vibration data refers to the vibration of the motor during the pump station startup process. Figure 9b is a schematic diagram of real-time vibration waveform detection through vibration detection. The vibration data of the motor includes two states: the static state and the normal operation state. The static state occurs before the “power start” activity, while the normal operation state occurs after it. Additionally, when the motor is in normal operation, its vibration data exhibits periodicity and high-frequency fluctuations within a certain threshold.

The temperature data and vibration data in the scene are formalized to facilitate the construction of the graph model. The activity of temperature represents the operating state of the temperature data, which is denoted as *T*(*i*), where *i* = {0, 1}.*T*(0) is the temperature outside the normal threshold range or the temperature range before the “Turn on the power” activity, and*T*(1) is the temperature range of the motor after “power on.” Vibration activity also represents the operating state of the vibration data, which is denoted as *V*(*i*), where *i* = {0, 1}. *V*(0) is the acoustic frequency of the sound wave that appears before the “power start” activity, and *V*(1) refers to the sound wave that appears after the normal acoustic frequency during the “power start.”

### 4.2 Graph model reconstruction

**Figure 10:**
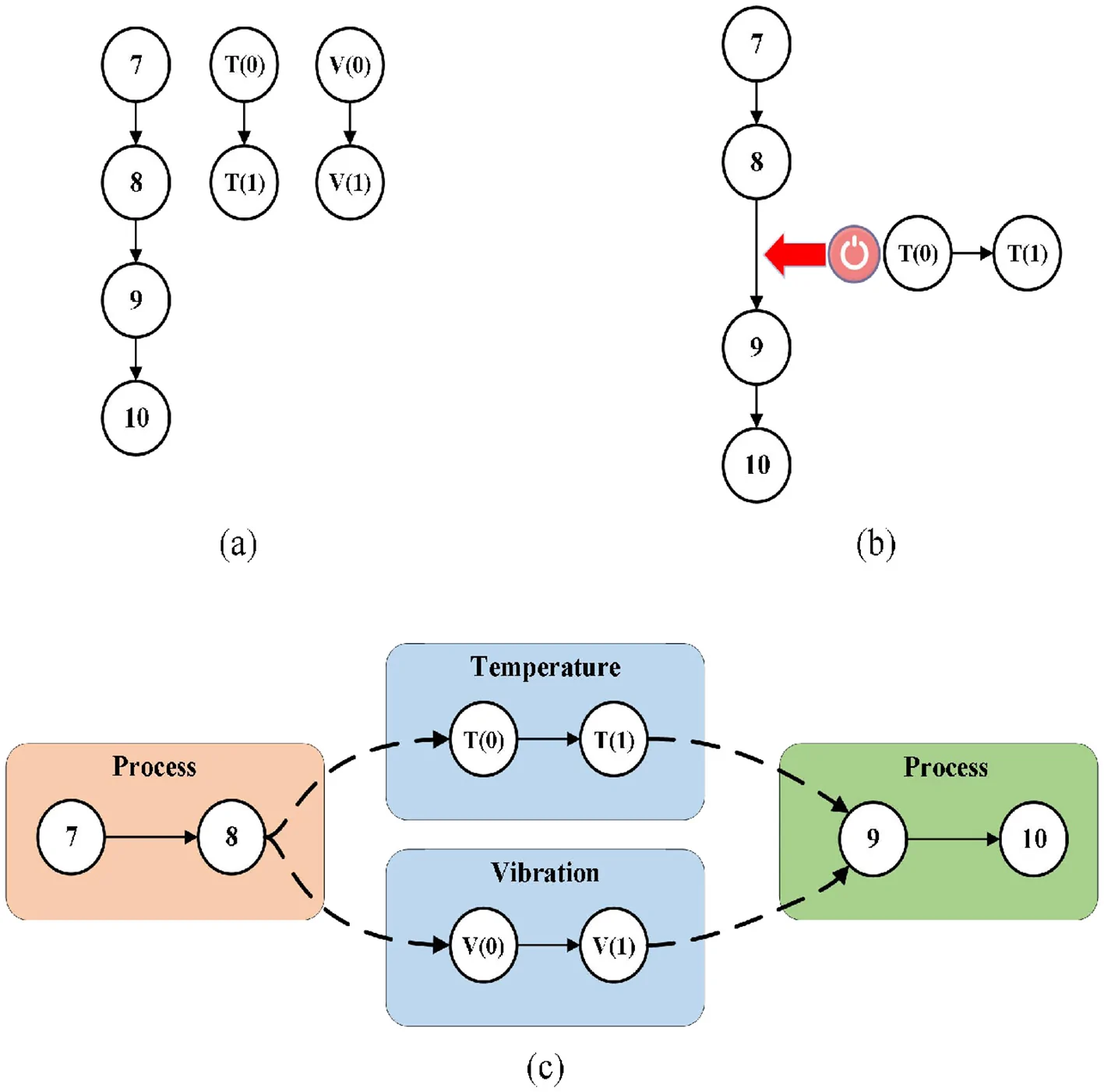
Business processes with business process activities integrated with external scenario activities.

The nodes and arcs in the graph are constructed by simulating the structure of the graph model. Nodes represent activities and arcs represent relational properties between activities, and the graph model can be represented by sets of nodes and activities. The graph model is defined as a tuple (*N, E*), where *N* is the set of active nodes and *E* is the set of arcs between activities. In this formalism, temperature and vibration information have two active nodes before and after startup, respectively, and the arcs between the nodes represent the relations of the startup power supply. In Figure 10a, the active traces of the pump station startup scenario and the traces consisting of external temperature and vibration are shown. The active node set and the arc set are also displayed. In the real scenario, the data for temperature and vibration changes when the power is activated. From the graph model, after the execution of active node 8, the active nodes T and V will also start to execute, as shown in Figure 10b. Therefore, it is necessary to add arcs to the graph to incorporate the traces of temperature and vibration into the traces of the pump station motor startup, as shown in Figure 10c.

To add the influence of sensor data on the pump station motor startup scenario to the log monitoring scope, discrete activities and process activities in the scenario were correlated. Activities 7 to 10 of a trace in the pump station startup process, including temperature and vibration traces, were selected. The activities in the trace are executed sequentially, and while these activities are running, the external temperature and vibration sensors monitor the operating data of the equipment synchronously. Detailed timestamps of these activities are recorded in the video data, and the sensor data includes the same timestamp information, which can be used to correlate the corresponding activities with the scene data through the link between the timestamps. The arcs in the arc collection E need to be redefined to associate new arcs. Add arcs (8, *T*(0))(8, *V*(0)), (*T*(1), 9) and (*V*(1), 9) and delete arcs (8, 9). By reconstructing the arc set e based on the actual scene activities, the reconstructed arc set*E* = {(7, 8), (8, *T*(0)), (8, *V*(0)), (*T*(0), *T*(1)), (*V*(0), *V*(1)), (*T*(1), 9), (*V*(1), 9), (9, 10)}, along with the reconstructed trace, is shown in Figure 10c.

The associated discrete data is added to the event log using Algorithm 6, and the fused event log includes discrete scene data for temperature and vibration. Algorithm 6 describes the fusion of scenario data for temperature and vibration into a pumping station startup business process log generated from video data. The input to this algorithm is log *L*, along with temperature and vibration data, and the output is the new log after fusion. The first step is to ensure that the temperature and vibration data are collected properly (refer to lines 1 to 2 of the algorithm). Since the time of the video stream coincides with the time of the external data sensors, the goal for each trace in the facilitated log is to find a matching timestamp. When the timestamps of the temperature and the activity in the video match, the temperature data is inserted into log L as an attribute column (refer to lines 3 to 7 of the algorithm), and the fusion of the vibration data proceeds in the same way (refer to lines 9 to 11 of the algorithm). Table 3 presents the enhanced event log of the second stage after associating scene data. This log includes the original log of the management system, as well as multimodal event logs that integrate video data and scene data.

Algorithm 6 **Discrete date completion.**

**Input**: Log:*L, T*(*i*), *V*(*i*)
**Output**: Log*:new L*
1. *Temperature*() ← Ø
2. *Audio*() ← Ø
3. **for** *L.case* **do**
4. *row* ← Ø
5. **if** *newL.Timestamp == T.date*() **then**
6. *row* ← *T*(*i*)
7. *L* ← *row*
8. **else**
9. *newL.Timestamp == T.date*()
10. *row* ← *V*(*i*)
11. *L* ← *row*
12. **end**
13. **end**
14. **return** new *L*

**Table 3:** Enhanced scene logs in the second stage.

Case	Act	Tool	Num	Position	Operator	S_t	E_t	C_t	Temp	VIB
e7	Check gauges	Helmet, gloves, and electroprobe	3	E	Mao	14:01:07	14:03:46	0:02:39	–	–
e8	Turn on the power	Helmet, insulated gloves	2	H	Mao	14:04:27	14:05:13	0:00:46	137	3.2
e9	Adjust flow rate	Helmet, gloves	2		Mao	14:06:02	14:09:32	0:03:30	137	3.2
e10	Checking the operating status	Helmet, gloves, and screwdriver	3		Mao	14:10:02	14:12:43	0:02:41	137	3.2

The current graph reconstruction mainly captures the temporal association between process activities and discrete sensor events. A natural extension is to represent the scenario simulation graph as a weighted directed graph, where edge directions indicate process precedence or sensor-to-activity influence, and edge weights quantify the strength of contextual impact. For example, a temperature fluctuation after power activation can be modeled as a directed edge from the temperature node to the related activity node, with the weight estimated from temporal overlap, signal magnitude, correlation with activity duration, or learned attention scores. This extension may further improve anomaly detection in highly dynamic industrial environments.

## 5 Experimentation and evaluation

In this section, the enhanced event log is used to detect anomalies in the pump station motor startup scenario, and a series of experiments are conducted to prove the effectiveness of the enhanced event log in complex scenarios. In terms of anomaly detection performance, mainstream detection algorithms are used for horizontal comparisons across multiple event log datasets. The characteristics of the enhanced scenario information event log in this study are demonstrated through actual data verification and comparative experiments. Additionally, ablation experiments are designed to compare the first-stage event log, the second-stage event log, and the original event log. The experiments not only record conventional indicators such as accuracy but also particularly focus on the model's stability performance when processing multi-source data. This systematic verification method, from data features to practical applications, can objectively evaluate the quality of the dataset and clearly demonstrate the improvement effect of the enhanced event log on anomaly detection capability in complex scenarios.

### 5.1 Artificial anomaly

**Figure 11:**
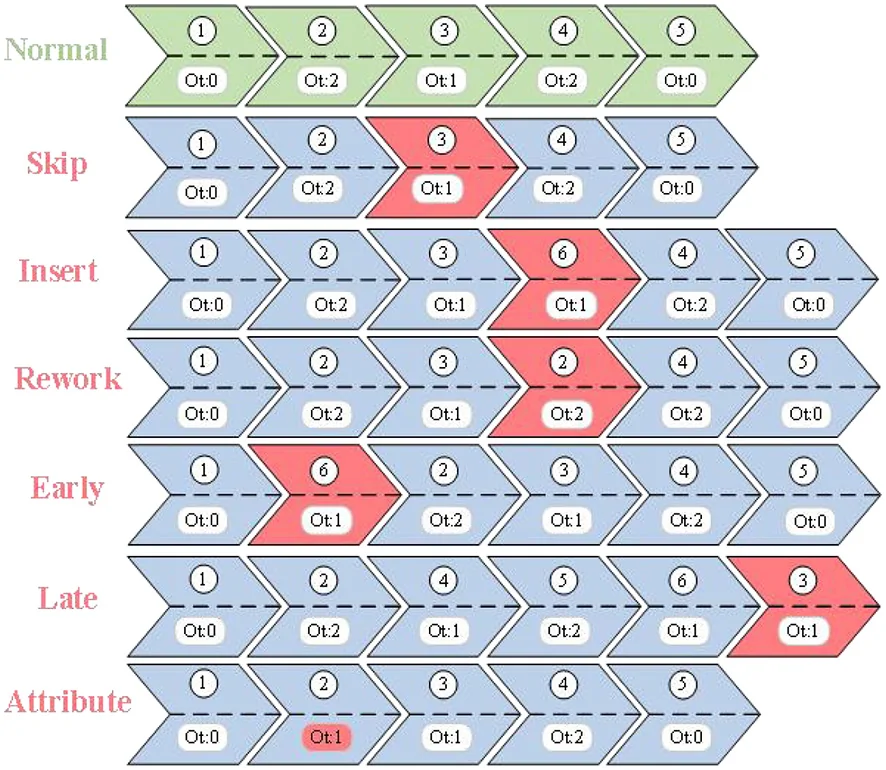
Different types of anomalies for normal traces during motor startup.

Due to a lack of anomaly data in event logs, anomalies need to be added to the event log dataset. In this study, we refer to Nolle et al. (2022), who injected artificial anomalies into event logs to perform anomaly detection on event log datasets. In the field of business flow mining, anomalies usually include trace anomalies and attribute anomalies, which are patterns of anomalies realized through temporal reorganization of event sequences and attribute value anomalies targeted to interfere with normal process traces. Therefore, six common types of anomalies are manually injected into the event log. Figure 11 illustrates the different anomaly traces obtained by applying the six types of anomalies to the normal traces. Among the six types of anomalies, skip, insert, refactor, advance, and defer are trace anomalies, while attribute anomalies involve incorrect values in the attributes. In Figure 11, green represents normal business process traces, blue represents business process traces with anomalies, and red indicates the abnormal parts. The sequence number indicates the order in which the business process traces are executed, and *Ot* indicates the included attribute value of the event. These exception types are defined below.

- Skip: skips a sequence of up to two events.
- Insert: a sequence of up to two random events is inserted.
- Reconstruct: sequences of up to three events are executed once more.
- Early: sequences of up to two events are executed too early and therefore later skipped.
- Deferred: sequences of up to two events are executed too late and therefore skipped ahead.
- Attribute: up to two attribute values are replaced with incorrect values in up to three events.

Before injecting artificial anomalies, we collected a baseline dataset consisting of 288 normal operational traces directly from a real-world pump station scenario. The experiments adopted a gradient anomaly load stress testing strategy, injecting anomaly proportions (AP values) ranging from 0.05 to 0.45 into these baseline logs and constructing a set of synthetic samples containing 342 groups (9AP × 32 processes for the completed event logs = 288 groups, and 9AP × 6 scenarios for the original event logs= 54 groups). This progressive anomaly intensity design effectively simulates a continuous anomaly environment, ranging from minor to systematic anomalies, and provides a quantitative benchmark for the scalability assessment of detection models.

In performing anomaly detection, a threshold τ is defined to categorize the anomaly score into two types: 0 or 1, where 0 indicates normal and 1 indicates an anomaly. The threshold can be determined by selecting it based on the actual environment or by using an existing threshold selection method. A threshold τ is necessary because the criteria for abnormality detection change under the influence of situational factors. For example, the temperature of the motor fluctuates during summer and winter, and by setting a threshold τ, the influence of the environment on anomaly detection can be reduced. An attribute value is marked as anomalous if its anomaly score exceeds the threshold τ (i.e., at the attribute level). The likelihood of an attribute value being marked as anomalous rises in direct proportion to its anomaly score.

Here, it is necessary to compute the anomaly score, which is based on anomaly identification. *e* is the trace, *t* is the anomaly on the trace and *a* is the anomaly on the attribute. The abnormal scores are given by Equations 9 and 10.


Se,t,a=∑1<i<Netia (9)

$$\begin{array}{l} L_{e,t,a}=\left\{\begin{array}{c} 1\\ 0 \end{array}\begin{array}{c} if\\ if \end{array}\begin{array}{c} S_{e,t,a}>\tau \\ S_{e,t,a>}\leq \tau \end{array}\right\} \end{array}$$ (10)

Consider, for example, a threshold of τ = 0.8T and a given anomaly score of τ = 0.75S, which is below 0.8. We conclude that the associated labeling of the attribute *S*_*e,t,a*_ of the event *L*_*e,t,a*_ in the trace τ is τ = 00, indicating normal. The following rule can be used for detecting anomalies that will be adapted to the trace level: if any attribute of any event in any trace is considered anomalous, then the whole trace is labeled as anomalous. The Equation 11 are calculated as follows:


$$\begin{array}{l} L_{e}^{trace}=\{\begin{array}{c} 1\\ 0 \end{array}\begin{array}{c} if\\ if \end{array}\begin{array}{c} \exists L_{e,t,a}=1\\ \forall_{e,t,a}=0 \end{array}\begin{array}{c} for\\ for \end{array}\begin{array}{c} e\in N\\ e\in N \end{array} \end{array}$$ (11)

### 5.2 Independent evaluation of IMFM-CMEA

Because the IMFM-CMEA module is the core component for cross-modal entity alignment in our two-stage enhancement method, it is essential to evaluate its performance independently of the downstream anomaly detection tasks. We assessed the module's capability using standard image–text retrieval and entity matching metrics, including Mean Reciprocal Rank (MRR), Recall@K (*K* = 1, 5), and Entity Matching Precision and Recall.

**Figure 12:**
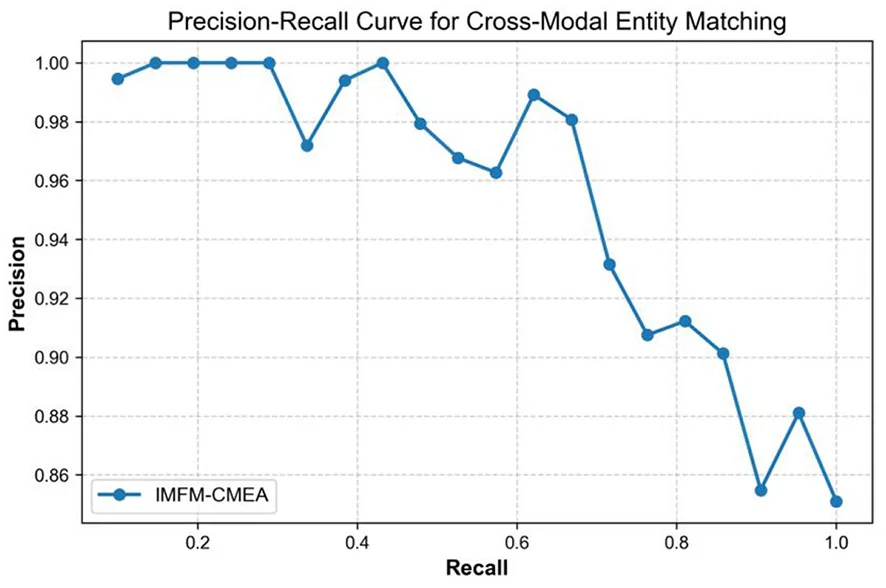
Precision–recall curve for entity matching.

For the entity matching task within the pump station motor startup scenario, the IMFM-CMEA module achieved an overall matching precision of 92.5% and a recall of 89.3%. As illustrated in the precision–recall (PR) curve (Figure 12), the module maintains a high level of precision even as recall increases, demonstrating its robustness in aligning discrete visual entities (Tool, Operator, Position) with text attributes. Furthermore, in the cross-modal retrieval evaluation, our method recorded an MRR of 0.87, with Recall@1 and Recall@5 reaching 88.5% and 96.2%, respectively.

**Figure 13:**
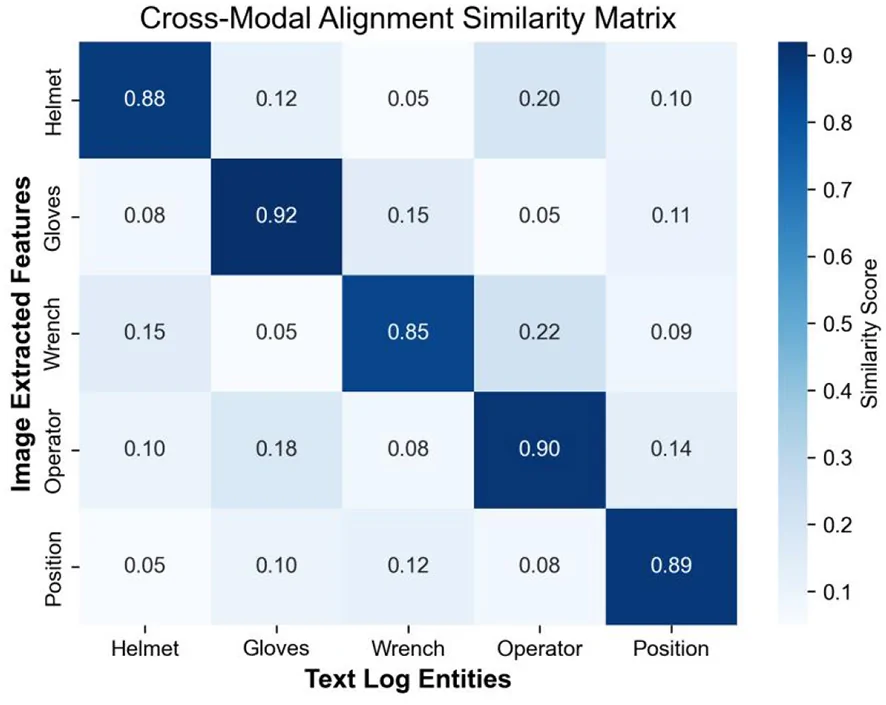
Similarity heatmap for cross-modal feature alignment.

To further visualize the effectiveness of the cross-modal feature alignment, Figure 13 presents the cosine similarity matrix between the image-extracted features and the text log entities. The correct matching pairs along the diagonal (Helmet, Gloves, Wrench, Operator, and Position) exhibit remarkably high similarity scores of 0.88, 0.92, 0.85, 0.90, and 0.89, respectively. Conversely, the off-diagonal scores for mismatched entities remain exceptionally low (mostly below 0.15). This stark contrast confirms that the gated multi-fusion module and the cross-modal contrastive learning strategy successfully capture fine-grained semantic connections, accurately distinguishing different scene targets and establishing a tight semantic alignment between unstructured scene images and structured logs before anomaly detection takes place.

### 5.3 Comparison model

To validate the advantages of the proposed method, we conducted a validation of business process anomaly detection performance by comparing it against multiple state-of-the-art methods on enhanced event logs and mainstream datasets. The mainstream datasets include Helpdesk (Verenich, 2016), Production, BPIC2017 (Van Dongen, 2015), BPIC2012, BPIC2012_O, BPIC2012_W (Van Dongen, 2012), and enhanced event logs. The selection of anomaly detection methods is based on the different types of datasets, and a variety of anomaly detection algorithms are chosen for comparison and detection. The specific methods are as follows (Guan et al., 2023, 2024a,b):

- DAE (Ko and Comuzzi, 2021): A trace feature embedding space is constructed using denoising auto-encoder (DAE) and variational auto-encoder (VAE) to quantify the degree of anomalies through potential representational reconstruction errors.
- BINet (Ko and Comuzzi, 2021): Identifies process deviation anomalies by building event attribute prediction models based on the Transformer architecture and utilizing timing prediction bias.
- VAE (Nolle et al., 2018): Converts traces to vector representations and detects anomalies based on the reconstruction error of a variational self-encoder.
- LAE (Rahmawati et al., 2016): Converts traces to vector representations, designs an LSTM-Autoencoder network to capture long-range dependency features, and evaluates anomalies by the degree of discrepancy between the decoder output and the original input.
- OC-SVM (Huo et al., 2021): Converts traces to vector representations and applies a class of SVMs (Verenich, 2016) to construct decision boundaries after mapping the traces to a heterogeneous feature space.
- Naive (Bezerra and Wainer, 2013): Assumes the Poisson distribution of the probability of occurrence of traces and applies a threshold filter to the sequence of low-frequency events.
- Sampling (Chandola et al., 2012): Detects anomalies by mining the reference model with the Alpha algorithm and calculating a compliance score for trace-model alignment.
- GAE (Schölkopf et al., 1999): Converts event logs into graph networks and detects topological anomalies based on edge reconstruction errors from the graph attention mechanism (GAT).
- DAE (Schölkopf et al., 1999): A denoising autoencoder (DAE) and a variational autoencoder (VAE) will be used to construct the trace feature embedding space, respectively, and quantify the degree of anomalies through the reconstruction error of potential representations.

### 5.4 Evaluation indicators

In this study, we use industry-popular *F* − *score* metrics to evaluate the performance of these methods, which include the average of *Precision* and *Recall*. The calculation equations for the indicators 12–14 are as follows:


$$\begin{array}{l} Precision=\frac{TP}{TP+FP} \end{array}$$ (12)

$$\begin{array}{l} Recall=\frac{TP}{TP+FN} \end{array}$$ (13)

$$\begin{array}{l} F-score=\frac{2*Precision*Recall}{Precision+Recall} \end{array}$$ (14)

A dynamic threshold optimization strategy is used for anomaly detection evaluation, A dynamic threshold optimization strategy is used for anomaly detection evaluation, making it crucial to choose an appropriate threshold τ when calculating the *F* − *score*. Without arbitrarily fixing τ, the*F* − *score* distribution curve is generated based on the anomaly scores of each sample, and the largest *F* − *score*is selected to be determined, where *TP*, *FN*, and *FP* denote true positives, false negatives, and false positives, respectively, by selecting the peak of the curve as the final performance metric. This method avoids the subjectivity of setting fixed thresholds and ensures that different methods are fairly compared under their respective optimal critical conditions, thus objectively reflecting the model's ability to detect different types of anomalies.

In Table 4, the anomaly detection performance of various methods at the business process trace level and attribute value level on the enhanced log Ours and each dataset is presented. The top three results are shown in bold, while the absolute best results are underlined. The following observations can be made: the experimental data show that the enhanced event log dataset can effectively support multi-dimensional anomaly detection. At the process activity level, the average of mainstream methods reaches over 0.85, with the attribute anomaly detection achieving an average value as high as 96%, verifying the reliability of the scenarios in terms of unstructured information added to the log. In trace and attribute-level anomaly detection, the enhanced event log maintains stable performance across all models, further confirming the advantage of the dataset in multi-source data alignment. These findings not only affirm the quality of event logs that incorporate scenario information but also reveal the matching patterns between different algorithmic features and business scenarios. It is worth noting that the performance of traditional anomaly detection methods shows significant differences, with OC-SVM lacking process context modeling capability and exhibiting the poorest detection results, whereas autoencoder-based schemes (DAE/VAE/LAE) are able to capture temporal features while focusing on the activities of the business process structure, resulting in higher anomaly detection accuracies. The BPIC2017 dataset ranks second in performance across all synthetic logs, mainly because the attribute values of the activities in the event log depend heavily on the relationships of preceding events, which better represent the dependencies.

**Table 4:** *F*-score on different logs, “T” and “A” for process and attribute anomaly detection.

Method	Logs													
	Helpdesk		Production		BPIC2017		BPIC2012		BPIC2012_O		BPIC2012_W		Ours	
	T	A	T	A	T	A	T	A	T	A	T	A	T	A
OC-SVM	**0.50**	–	0.48	–	**0.52**	–	0.45	–	0.48	–	0.45	–	**0.54**	–
Naive	**0.87**	–	0.85	–	**0.90**	–	0.69	–	0.72	–	0.69	–	**0.93**	–
Sampling	0.90	–	0.89	–	**0.90**	–	0.86	–	0.91	–	**0.89**	–	**0.92**	–
GAE	0.47	–	**0.56**	–	0.47	–	0.45	–	**0.53**	–	0.43	–	**0.87**	–
DAE	0.80	0.47	0.77	0.48	**0.83**	0.46	0.71	0.44	0.75	0.43	0.69	0.42	**0.89**	**0.97**
VAE	**0.83**	0.19	0.66	0.21	0.79	0.22	0.64	0.23	0.77	0.20	0.59	0.21	**0.85**	**0.93**
LAE	0.68	0.24	0.67	0.27	**0.75**	0.24	0.58	0.27	0.57	0.25	0.53	0.27	**0.90**	**0.95**
BINet	0.54	0.33	0.56	0.34	**0.57**	0.36	0.52	0.32	0.55	0.33	0.53	0.33	**0.87**	**0.98**

Bold font is used to indicate the optimal values in that column, and underlined font represents the optimal values across all experiments.

**Figure 14:**
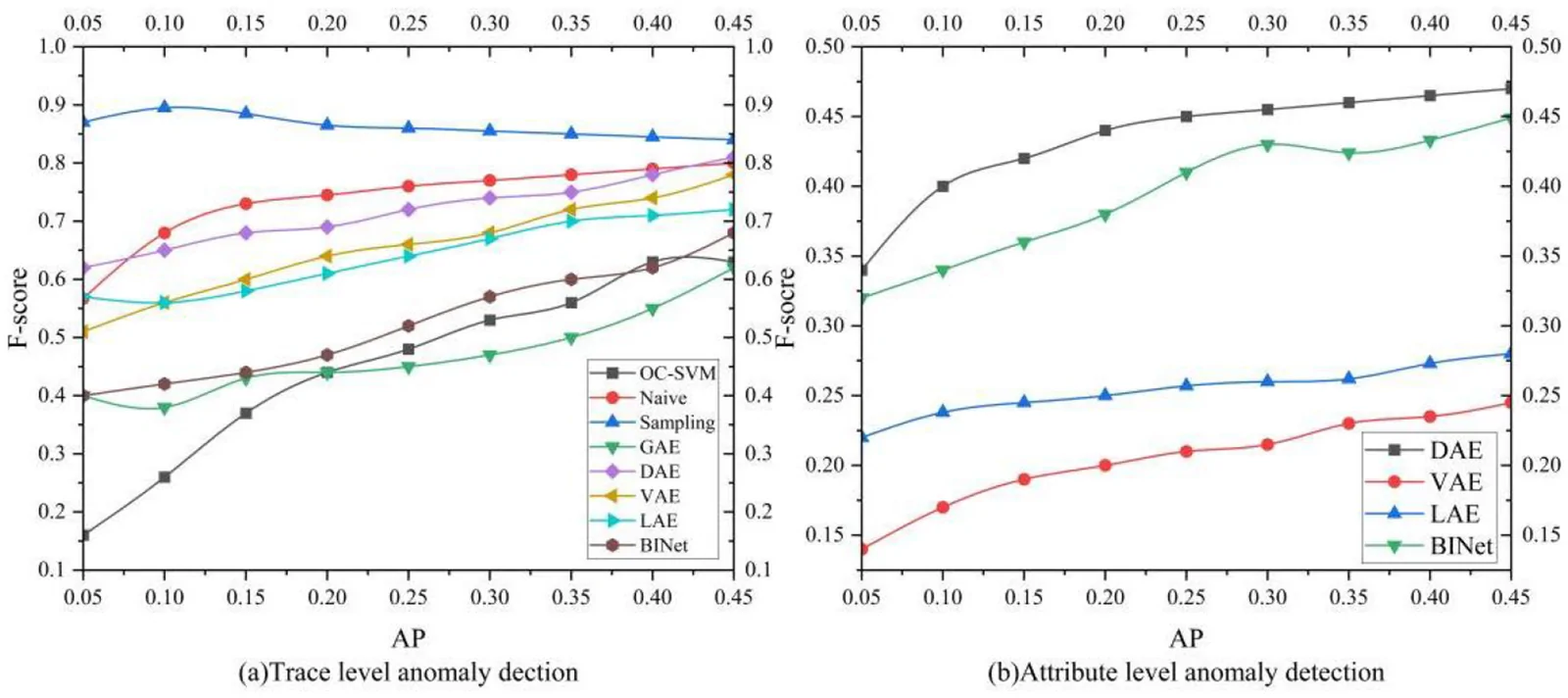
For the event log dataset under different APs. **(a)** Trace level anomaly detection. **(b)** Attribute level anomaly detection.

The experiments further explored the influence of the anomaly proportion (AP value) on the detection effect. As shown in Figure 14a, the Sampling method performs most stably in process step anomaly detection, consistently remaining around 0.89, which is not affected by fluctuations in AP values. In contrast, the other methods—OC-SVM, Naive, GAE, DAE, VAE, LAE, and BINet—show an upward trend with the AP value. This is mainly due to the fact that the higher the proportion of anomalous examples, the closer the accuracy of random guessing is to the preset AP value when the detection sensitivity is insufficient. Notably, in Figure 14b, the attribute anomaly detection scenario shows that the DAE model has a significant advantage, leading the other methods by about 15% at different AP values. Both types of detection models exhibit a commonality: when the AP value exceeds 0.3, the performance fluctuation of all models tends to level off, indicating that the dataset maintains detection stability under a high-pressure testing environment.

### 5.5 Ablation experiment

To verify the impact of scene information on the detection of business process anomalies, ablation experiments were designed to test the original log dataset of the management system and the log dataset enhanced by scene videos. Anomaly detection of process traces and attribute values was conducted on both types of logs, still using eight anomaly detection models. Original represents the original log dataset of the management system, and First represents the log dataset enhanced in the first stage. The top three best results are highlighted in bold, while the absolute best result is underlined.

Through the experiments in Table 5, it was found that the horizontal comparison of the scenario event logs at different enhancement stages with the mainstream event log datasets verified the completeness and validity of the scenario information. In process step anomaly detection, the Original dataset, which only contains trace information and lacks attribute information, can only perform trace anomaly detection. Due to the absence of event attribute information, the Original dataset exhibits poor anomaly detection performance. The dataset enhanced in the second stage supplemented the event attribute information in the log using scene video information, achieving better anomaly detection effects in both trace and attribute aspects. Compared with the Original dataset, the anomaly detection*F* − *score* improved by 70%. However, the Video dataset lacks the supplementation of discrete information in the scene, and its anomaly detection effect *F* − *score*decreased by 8% compared with the enhanced event log.

**Table 5:** *F*-score presents the results of the ablation experiments, where “T” and “A” stand for anomaly detection of processes and attributes.

Method	Logs																	
	Helpdesk		Production		BPIC2017		BPIC2012		BPIC2012_O		BPIC2012_W		Original		First		Ours	
	T	A	T	A	T	A	T	A	T	A	T	A	T	A	T	A	T	A
OC-SVM	0.50	–	0.48	–	**0.52**	–	0.45	–	0.48	–	0.45	–	0.13	–	**0.76**	–	**0.54**	–
Naive	**0.87**	–	0.85	–	**0.90**	–	0.69	–	0.72	–	0.69	–	0.21	–	0.86	–	**0.93**	–
Sampling	0.90	–	0.89	–	**0.90**	–	0.86	–	**0.91**	–	0.89	–	0.24	–	0.87	–	**0.92**	–
GAE	0.47	–	**0.56**	–	0.47	–	0.45	–	0.53	–	0.43	–	0.17	–	**0.74**	–	**0.87**	–
DAE	0.80	0.47	0.77	0.48	0.83	0.46	0.71	0.44	0.75	0.43	0.69	0.42	0.16	–	0.85	**0.87**	**0.89**	**0.97**
VAE	**0.83**	0.19	0.66	0.21	0.79	0.22	0.64	0.23	0.77	0.20	0.59	0.21	0.16	–	0.80	**0.83**	**0.85**	**0.93**
LAE	0.68	0.24	0.67	0.27	0.75	0.24	0.58	0.27	0.57	0.25	0.53	0.27	0.07	–	**0.83**	0.73	**0.90**	**0.95**
BINet	0.54	0.33	0.56	0.34	0.57	0.36	0.52	0.32	0.55	0.33	0.53	0.33	0.10	–	**0.82**	**0.82**	**0.87**	**0.98**

Bold font is used to indicate the optimal values in that column, and underlined font represents the optimal values across all experiments.

**Figure 15:**
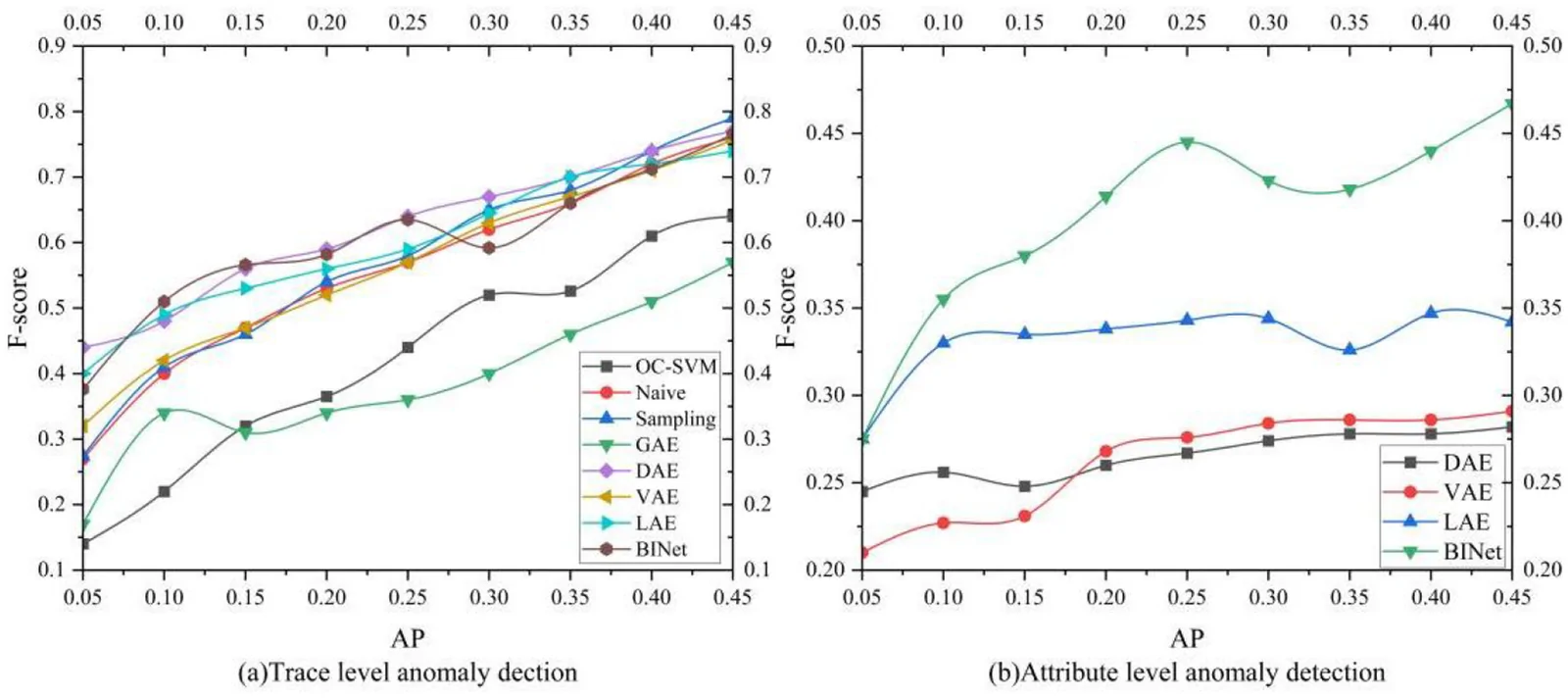
The figure shows event log dataset under different APs. **(a)** Trace level anomaly detection. **(b)** Attribute level anomaly detection.

The impact of the anomaly ratio AP value on detection effectiveness is investigated in Figure 15. The LAE and BINet approaches perform optimally in scenarios that do not contain scenario data. The advantage of BINet is even clearer when dealing with BPIC2017, which has a strong back-and-forth correlation but a short temporal dependency, precisely matching BINet's ability to model local features. It is worth noting that OC-SVM and GAE consistently perform poorly in all tests, which is directly related to their lack of business process context modeling. Further analysis of the impact of the anomaly percentage on scene detection without environmental data reveals that when the AP value rises from 5% to 45%, the process anomaly detection improves by an average of 28% across all methods, as shown in Figure 15a. There are two key factors behind this increase: first, there are fewer anomalies in the synthetic logs themselves that may have unlabeled environmental data, and with the injection of artificial anomalies from computer vision, the models can learn the anomaly patterns more thoroughly; second, the baseline accuracy of random detection naturally improves in a high AP-value environment. It is noteworthy that the Sampling method, which previously performed well on synthetic data, experienced a performance drop in synthetic scenarios (a decline of 12%), highlighting the challenge of implicit rules in real business processes on the generalization ability of the model. In contrast, at the level of attribute anomaly detection in Figure 15b, the performance of the models remains stable (fluctuating by 5%), confirming their robustness in multi-dimensional anomaly characterization.

### 5.6 Computational efficiency and deployment considerations

For practical deployment, computational efficiency is an important issue because the proposed framework involves video frame sampling, visual feature extraction, cross-modal entity matching, graph-based sensor fusion, and downstream anomaly detection. In the current implementation, the video stream is sampled at one frame per second to balance information completeness and processing cost. The most computationally expensive components are image preprocessing and VLP-based feature encoding, whereas timestamp-based sensor fusion can be implemented with near-linear complexity after indexing events and sensor observations. Future work will evaluate real-time latency under different hardware settings and explore lightweight visual backbones, feature caching, model quantization, and knowledge distillation to reduce computational costs while preserving detection accuracy.

### 5.7 Influence of τ parameters

**Figure 16:**
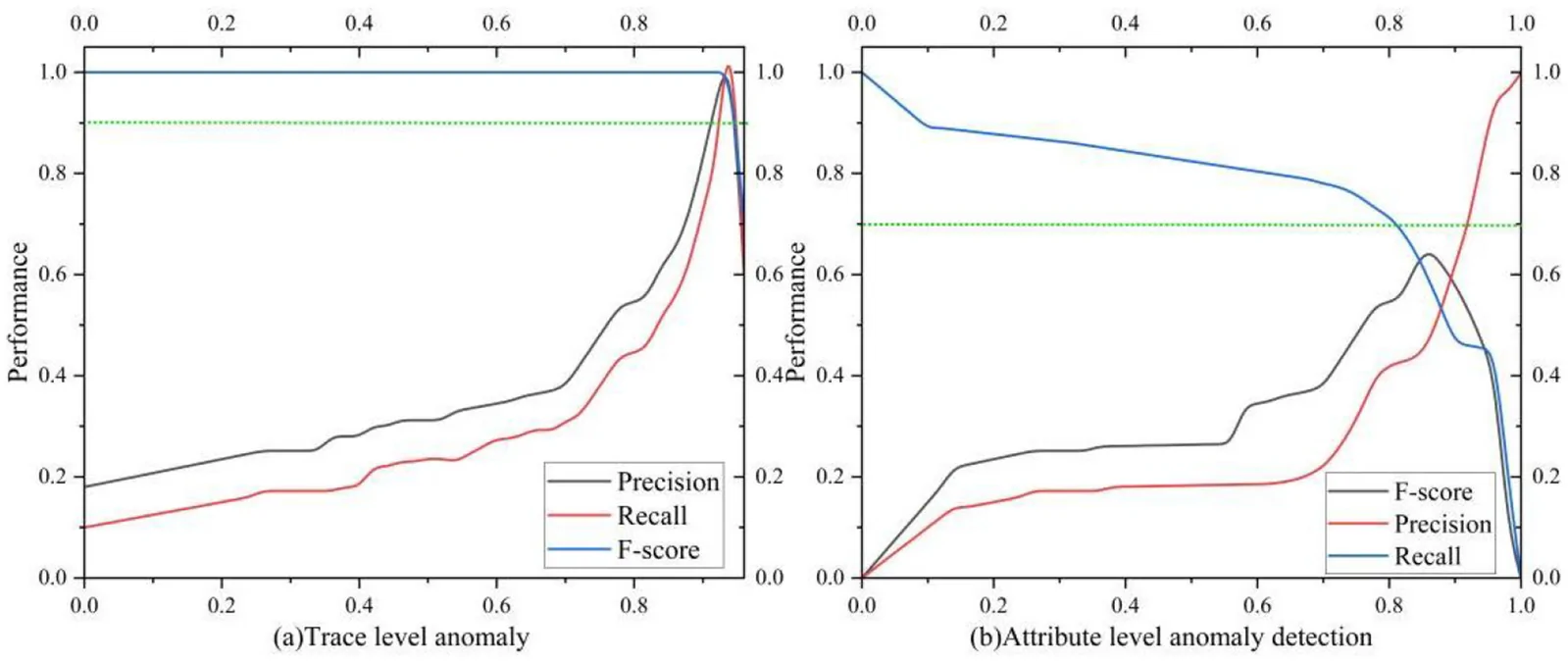
The figure of the event log dataset under different APs. **(a)** Trace level anomaly detection. **(b)** Attribute level anomaly detection.

In this study, we evaluate the dynamic impact of the threshold on the detection effect: as it gradually increases from 0, the anomaly labeling criterion tightens, resulting in a rise in accuracy accompanied by a simultaneous increase in the missed detection rate. As shown in Figure 16, in the test scenario with AP = 0.1, there is a parabolic trend that first increases and then decreases (the peak value is about 0.95). It is noteworthy that the methods demonstrate good parameter adaptation in completing the event log. When the threshold is in the range of 0.91 to 0.98, the detection rate of process step anomalies remains consistently higher than 0.9, and the attribute anomaly detection stabilizes above 0.7. The extreme test case shows that when the threshold is set to 0 (i.e., all labeled as anomalies), the accuracy is exactly equal to the preset anomaly proportion (0.1), which aligns fully with theoretical expectations. These findings confirm that although threshold selection requires a trade-off between false alarms and missed detections, the robustness of the test model ensures reliable detection over a wide range of parameters.

## 6 Conclusion

The main focus of this study is to propose a two-stage method for enhancing event log information, which takes into account the correlation between unstructured data in the scene and structured logs from the management system. By matching and aligning equivalent entities across different modalities, the proposed method enhances scene event logs and improves the ability of business process anomaly detection to identify abnormalities that are not visible in traditional management-system logs. Based on the experimental results, the method proposed in this study demonstrates effectiveness and superiority in business process anomaly detection. However, some anomalies were not successfully detected, mainly trace-level anomalies. After analysis, we speculate that two factors may contribute to this situation. First, the relationships between activities have not been thoroughly explored, which may overlook possible dependencies between different activities. Second, the amount of collected scene data is limited, and other unattended contextual information may still affect the anomaly detection results. Therefore, it is necessary to expand the collection of new data and further investigate the dependencies between data in future work. Nevertheless, several limitations remain. First, the experimental validation is currently limited to the pump station motor startup scenario. Although the proposed two-stage enhancement framework is designed for general complex process environments with incomplete structured logs and additional visual or sensor information, its applicability to healthcare workflows, logistics operations, and broader manufacturing scenarios still requires further empirical verification. Second, the current scenario simulation graph mainly models temporal associations between process activities and discrete sensor events. In future work, we will extend it to a weighted directed graph so that the direction and strength of sensor-to-activity influence, such as the impact of temperature fluctuations on activity duration, can be explicitly represented. Third, practical industrial deployment requires a more systematic evaluation of computational efficiency. We will further investigate real-time video processing latency, lightweight VLP backbones, feature caching, quantization, and knowledge distillation to improve scalability while maintaining anomaly detection accuracy. In addition, multi-modal collaborative fusion, spatio-temporal graph neural networks (STGNN), and knowledge graphs will be considered to enhance the cross-domain adaptability and robustness of the proposed framework.

## Data Availability

The raw data supporting the conclusions of this article will be made available by the authors, without undue reservation.
